# Commissioning and first-year operational results of the MAX IV 3 GeV ring

**DOI:** 10.1107/S1600577518008111

**Published:** 2018-08-27

**Authors:** Pedro F. Tavares, Eshraq Al-Dmour, Åke Andersson, Francis Cullinan, Brian N. Jensen, David Olsson, David K. Olsson, Magnus Sjöström, Hamed Tarawneh, Sara Thorin, Alexey Vorozhtsov

**Affiliations:** a MAX IV Laboratory, Lund University, PO Box 118, SE-22100 Lund, Sweden

**Keywords:** storage ring, synchrotron light source, multibend achromat, MAX IV

## Abstract

The MAX IV 3 GeV electron storage ring in Lund, Sweden, is the first of a new generation of light sources to make use of the multibend-achromat lattice (MBA) to achieve ultralow emitance and hence ultrahigh brightness and transverse coherence. The conceptual basis of the MAX IV 3 GeV ring project combines a robust lattice design with a number of innovative engineering choices: compact, multifunctional magnet blocks, narrow low-conductance NEG-coated copper vacuum chambers and a 100 MHz radio-frequency system with passively operated third-harmonic cavities for bunch lengthening. In this paper, commissioning and first-year operational results of the MAX IV 3 GeV ring are presented, highlighting those aspects that are believed to be most relevant for future MBA-based storage rings. The commissioning experience of the MAX IV 3 GeV ring offers in this way an opportunity for validation of concepts that are likely to be essential ingredients of future diffraction-limited light sources.

## Abstract 2

Commissioning and first-year operational results of the MAX IV 3 GeV electron storage ring, the first synchrotron light source to make use of the multibend-achromat lattice to achieve ultralow emittance and high brightness, are presented.

## 1. Introduction   

The MAX IV 3 GeV ring in Lund, Sweden (Tavares *et al.*, 2014*a* ▸), is the first of a new generation of synchrotron light sources (Hettel, 2014 ▸) which employ a multibend-achromat lattice (Einfeld & Plesko, 1993 ▸) to reach emittances in the few hundred pm rad range in a circumference of a few hundred metres, with the aim to enable the realization of new classes of experiments (Eriksson *et al.*, 2014 ▸), which are critically dependent on source brightness and transverse coherence.

**Figure 1:**
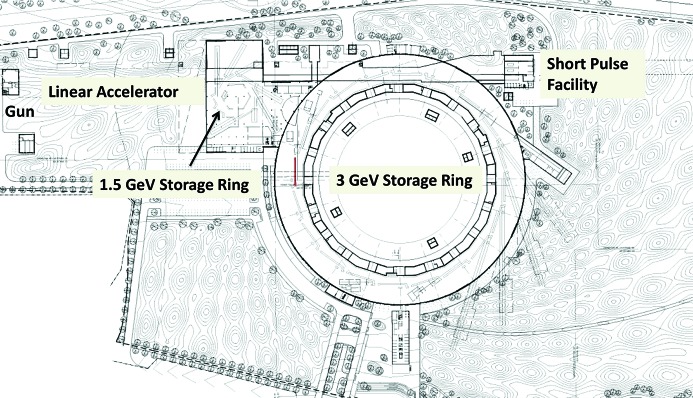
Overview of the MAX IV facility.

The core of the MAX IV facility [see the MAX IV detailed design report (MAX IV, 2010 ▸)] (Fig. 1 ▸) consists of three electron accelerators and their respective synchrotron radiation beamlines. Two electron storage rings operate at different energies (1.5 GeV and 3 GeV) in order to cover a wide photon energy range in an optimized way with short-period insertion devices, whereas a linear accelerator acts as a full-energy injector into both rings and provides electron pulses as short as 100 fs to produce X-rays by spontaneous emission in the undulators of the short-pulse facility.

The 3 GeV ring is optimized for the production of high-brightness hard X-ray beams and features a 20-fold seven-bend achromat lattice (Leemann *et al.*, 2009 ▸), reaching a bare lattice emittance of 0.33 nm rad. Achieving such a low emittance in only 528 m of circumference requires a compact magnet design (Johansson *et al.*, 2014 ▸) with small magnet gaps that allow the required integrated gradients to be achieved within short lengths as well as short distances between consecutive magnets. Moreover, these compact magnets are built as integrated units in which the bending-magnet poles and quadrupole pole roots are machined out of a pair of solid-iron blocks, which are assembled together, each unit holding all the magnets of a complete cell. As a result, the alignment accuracy within a cell is defined by machining and assembly accuracy rather than magnetic fiducialization procedures. Moreover, high natural vibration frequencies of the units are achieved, thus reducing the sensitivity of the magnets to the environmental vibrational noise.

The compact magnet design leads to narrow low-conductance vacuum chambers (Al-Dmour *et al.*, 2014 ▸) requiring distributed pumping and distributed absorption of the synchrotron radiation heat load. The choice of copper for the chamber material helps to alleviate the heat-load problem with chamber cooling being provided along the extended region over which the synchrotron radiation heat is deposited, whereas distributed pumping is provided by non-evaporable getter (NEG) coating of the chamber’s inner surface.

The reduced chamber dimensions lead in turn to an increased risk of collective instabilities (Tavares *et al.*, 2011 ▸), such as coupled bunch instabilities driven by the resistive wall impedance. A key ingredient in dealing with this problem is the use of passively operated third-harmonic cavities (Tavares *et al.*, 2013 ▸), which lengthen the bunches, reduce the electron density and help maintain the heat load from beam-induced fields on vacuum components at manageable levels. Moreover, they increase the incoherent synchrotron frequency spread, which provides Landau damping of coherent instabilities.

The 100 MHz radio-frequency (RF) system (Andersson *et al.*, 2011 ▸) uses capacitive-loaded normal conducting cavities, of the same type as previously developed for MAX II and MAX III. The relatively low RF facilitates achieving a large bucket height with relatively low RF voltage. The power can be obtained from reliable high-efficiency solid-state RF transmitters largely used in telecommunications, reducing investment and operation costs. In addition, the cavity design pushes the frequencies of the first higher-order modes (HOMs) of the cavity to about four times the fundamental mode frequency, limiting the overlap of the cavity impedance spectrum with the spectrum of the lengthened bunches.

Beam orbit stability is of paramount importance to preserve the performance of the source. Careful design of the floor as well as of all mechanical support structures in the accelerator and beamlines coupled with a permanent vigilance to prevent new mechanical noise sources from being introduced are crucial to maintain, as much as possible passively, the stability of the photon beam.

The engineering design choices described above constitute an integrated solution to the implementation of the MBA concept and are the result of a global optimization process in which the overall source performance, standardization and modularity were overarching guiding principles.

**Figure 2:**
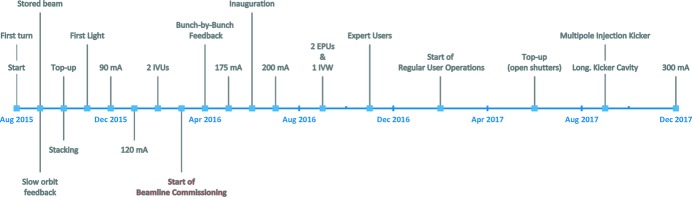
MAX IV 3 GeV ring commissioning and operation timeline. IVUs, IVW and EPUs refer, respectively, to in-vacuum undulators, in-vacuum wiggler and elliptically polarizing undulators. More details can be found in the references cited in the text.

A description of the early MAX IV 3 GeV ring commissioning efforts is given by Eriksson *et al.* (2016 ▸) and Tavares *et al.* (2016 ▸) whereas first optics and beam dynamic studies are reported by Leemann *et al.* (2018 ▸). Fig. 2 ▸ summarizes the main commissioning and operation milestones as well as the accelerator performance evolution since the start of commissioning and up to the end of 2017.

The design horizontal emittance was confirmed experimentally (Andersson *et al.*, 2016 ▸) and a vertical emittance down to 3 pm rad was demonstrated. Up to 300 mA (multibunch) and 9 mA (single-bunch) was stored in the ring and the product of beam current and lifetime reached 7 A h after 160 A h of accumulated beam dose. Injection efficiencies in excess of 90% with transient perturbations below ±13 µm (horizontal) and ±8 µm (vertical) have been demonstrated with a multipole injection kicker. Excellent beam position stability, even without a fast orbit feedback system, was achieved. Measurements with beam indicate that the beam coupling impedance was underestimated, but relevant single-bunch instability thresholds are still far above the nominal bunch current. Multibunch instabilities, in particular longitudinal coupled bunch modes driven by main and harmonic cavity HOMs, have shown to be the most troublesome to overcome and a bunch-by-bunch feedback system was implemented to damp those instabilities. The harmonic cavities were successfully used to provide lengthening by up to a factor of two.

In this paper, we describe commissioning and first-year operational results of the MAX IV 3 GeV ring, focusing on the latest achieved performance and highlighting those aspects that we believe are most relevant for future MBA-based storage rings. §2 lists the achieved results of the MAX IV linac with a focus on its performance as an injector to the MAX IV 3 GeV ring. §3 lists recent developments on characterization and trimming of the ring optics, which update and complement the initial results reported by Leemann *et al.* (2018 ▸). §4 reports both single-bunch and multibunch experimental coherent collective effects studies and §5 describes the overall strategy for achieving a stable beam orbit at MAX IV as well as the specific results obtained at the MAX IV 3 GeV ring. The following sections report on performance and operational experience with transparent top-up injection (§6) and the magnet (§7), vacuum (§8), RF (§9) and insertion device (§10) systems, whereas §11 summarizes first-year reliability statistics. Finally, §12 presents conclusions and future plans for further improvements and upgrades.

## 2. The MAX IV injector   

**Figure 3:**
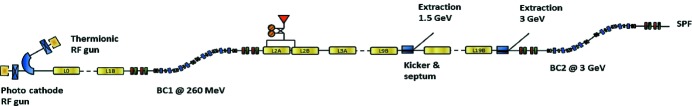
Layout of the MAX IV linac. The beam is extracted for ring injection at 1.5 and 3 GeV.

The MAX IV injector (Fig. 3 ▸) consists of two electron guns and a 250 m-long 3 GeV linear accelerator. For injection and top-up to the storage rings, a thermionic gun with a chopper system, delivering a train of ten 100 MHz RF bunches, is used (Olsson *et al.*, 2014 ▸). In high-brightness mode, delivering low-emittance 100 fs bunches to a short-pulse facility (Werin *et al.*, 2009 ▸), we use a 1.6 cell photo-cathode gun capable of producing an emittance of 0.4 mm mrad at a charge of 100 pC (Andersson *et al.*, 2017 ▸). This photo-cathode gun has also been commissioned to inject beam into the storage rings, hitting a single ring RF bucket at each shot. Photo-gun injection has been successfully tested, albeit without phase lock between laser shot and ring RF. A coincidence detector system is being implemented that will allow injection to be trigged exactly when the timing of the laser pulse and ring RF bucket coincide.

Acceleration is performed in 39 warm S-band linac sections driven by 20 RF units, each consisting of a 35 MW klystron and a solid-state modulator. The klystrons are operated at the lower power of 25 MW which reduces the operational cost and gives a total redundancy in energy of 0.6 GeV. The energy gain is increased with SLED cavities (Farkas *et al.*, 1974 ▸).

The three first RF units are driven individually by a low-level RF system, whereas a small fraction of the power from the last one of these is used to feed the main drive line (MDL) that provides input RF power to the remaining 17 RF units. The RF phase can be set individually in the first three units and power can be set individually for each RF unit. The MDL is situated inside the linac tunnel so that tunnel temperature variations affect both the linac structures and the MDL in the same way, keeping the relative phase between electrons and the accelerating RF waves essentially constant.

The beam is extracted for injection into the storage rings at 1.5 and 3 GeV. To meet exactly the right energy required for each ring, the fill-time to the energy doubling SLED units is varied. This instantaneously sets the right energy at a specific point in the linac. At the moment, both rings are injected with a repetition rate of 2 Hz, but work to obtain radiation safety permission for the design value of 10 Hz is ongoing. Table 1 ▸ shows the current status of some beam parameters for the linac in ring injection mode.

## 3. Bare lattice optics characterization and trimming   

**Figure 4:**
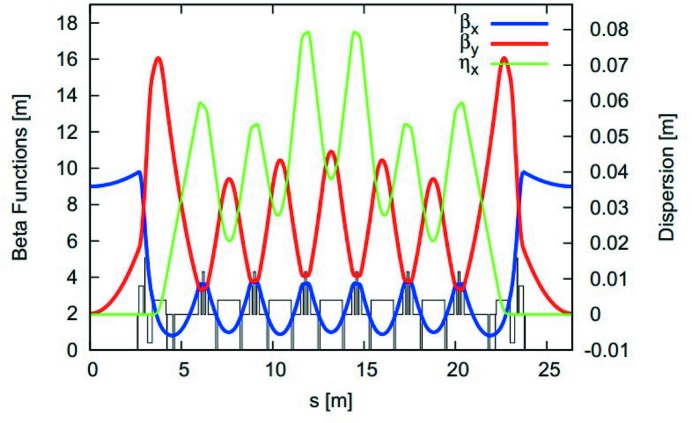
MAX IV 3 GeV ring optics functions (MAX IV, 2010 ▸).

We now move on to the main subject of this paper, namely, the 3 MAX IV 3 GeV ring and start by reporting on the characterization of the electron beam optics without insertion devices. The seven-bend-achromat lattice and its resulting optics functions can be seen in Fig. 4 ▸, whereas the lattice-related parameters are given in Table 2 ▸. Before any attempt to characterize the lattice, it is of highest importance to correct the orbit so that the beam crosses the various lattice elements at their magnetic centres. This is particularly important for sextupole magnets as an off-centre beam in a sextupole magnet leads to perturbations to the transverse focusing. An appropriate orbit correction requires in turn that the offsets of the beam position monitor (BPM) centres with respect to the magnetic centres of adjacent magnets be determined accurately. This procedure was partly described by Leemann *et al.* (2018 ▸). However, a puzzling fact was that the BPM offsets seemed to depend on the excitation strength of the magnet in question. The root cause has been traced down to iron saturation and a detailed explanation is given in §7.

For characterization and trimming of the linear part of the lattice optics, the most accepted method is LOCO (linear optics from closed orbits) (Safranek, 1997 ▸). This method was extensively used during commissioning and first year of operation of the MAX IV 3 GeV ring. The procedure and the results as of June 2017 are described by Leemann *et al.* (2018 ▸). Since then the work on the linear lattice has largely been focused on reducing coupling and automating the optics correction procedure, enabling operators to perform it routinely. In order to achieve the latter it proved necessary to reduce the number of gradient knobs available to LOCO as the reported solutions otherwise frequently requested larger adjustments of the dipole gradients than could be provided by the pole face strips. To avoid this, all dipole gradients were set to the nominal settings according to field map data and then removed from the LOCO fit procedure. At the same time, skew quadrupole knobs were added to enable simultaneous correction of coupling and vertical dispersion. For this, the trim coils configured in skew quadrupole mode in the OXX (octupole) and SXFO (sextupole) magnets were used.^1^ Initially this was not successful, due to the applied skew fields not matching the measured calibration curves. In the case of the SXFO family, the observed mismatch could be explained by iron saturation, stemming from the main coil excitation, in a similar way to the aforementioned effects of iron saturation on measured BPM offstes. In the case of the OXX, the skew field seen by the beam was stronger than expected, indicating another error source. In both cases, family correction factors had to be determined empirically in order to compensate. Work is ongoing to include a general saturation compensation for the trim coils in the matlab-middlelayer (MML) (Portmann *et al.*, 2005 ▸), based on magnetic simulation data.

**Figure 5:**
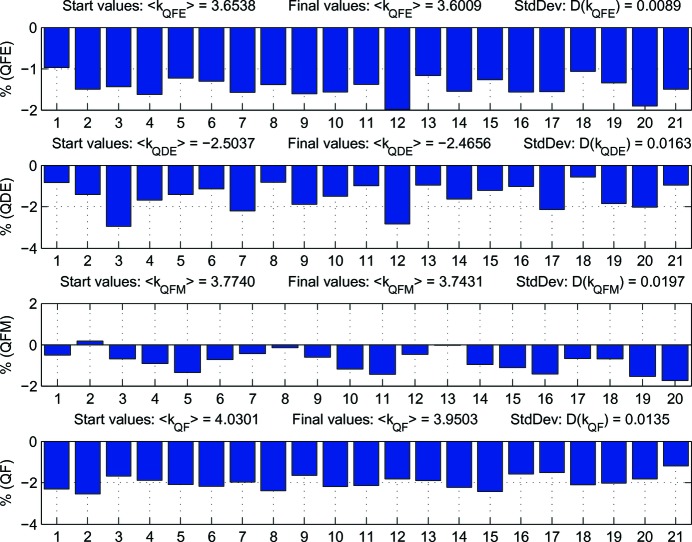
Relative quadrupole gradient changes required by the LOCO algorithm.

**Figure 6:**
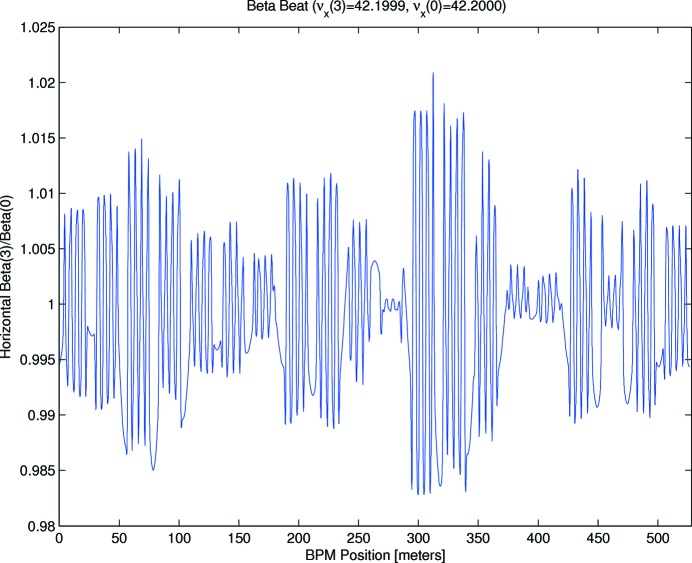
Residual horizontal beta-beat after implementing gradient changes given in Fig. 5 ▸ and the additional skew quadrupole corrections.

**Figure 7:**
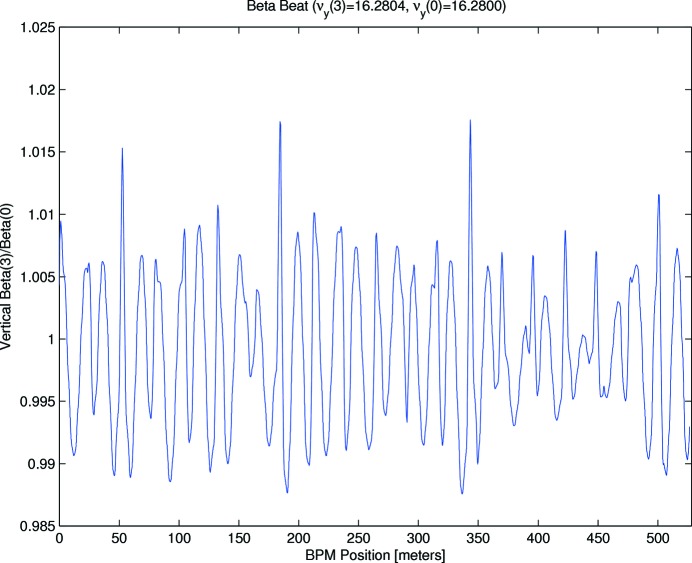
Residual vertical beta-beat after implementing gradient changes given in Fig. 5 ▸ and the additional skew quadrupole corrections.

**Figure 8:**
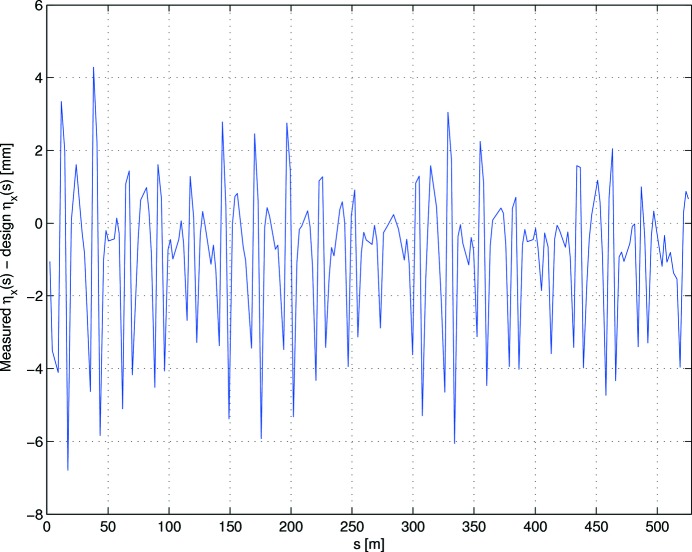
Residual deviation from design of the horizontal dispersion function after implementing gradient changes given in Fig. 5 ▸ and the additional skew quadrupole corrections.

**Figure 9:**
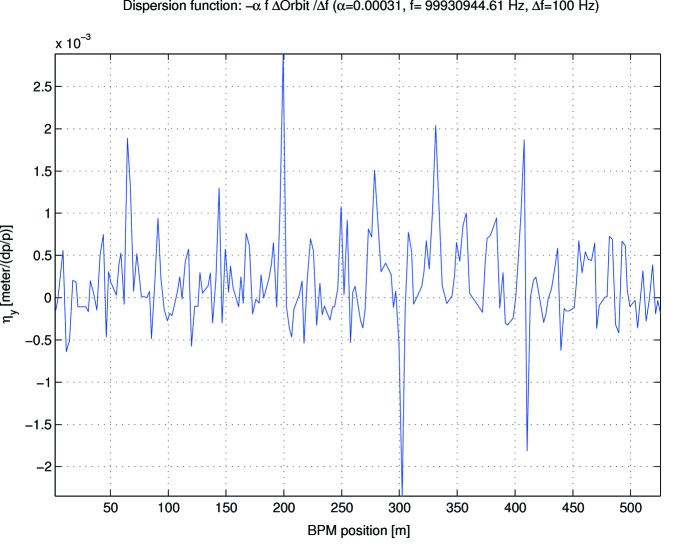
Residual vertical dispersion function after implementing gradient changes given in Fig. 5 ▸ and the additional skew quadrupole corrections.

Once an optics correction campaign using the above fit strategy had finished, an additional LOCO measurement was taken to evaluate the result, which is summarized in Figs. 5–9. Fig. 5 ▸ shows the gradient adjustments relative to theoretical settings derived from the measured excitation curves. As can be seen, adjustments of up to 3% were required and there were shifts in the mean gradients, which are significantly larger than the results of Leemann *et al.* (2018 ▸). Unlike them, the two global gradient knobs for the DIPM (40 magnets in series) and DIP (100 magnets in series) have not been adjusted from nominal settings. As there is one gradient dipole between every BPM pair apart from the straights, the mean gradients in the other families would have to be adjusted by LOCO to compensate. It should be noted that the gradient spread within the families is significantly lower, ranging from a minimum of 0.25% RMS for QFE to a maximum of 0.66% RMS for QDE.

Figs. 6 ▸ and 7 ▸ show the remaining horizontal (3.8% peak-to-peak, 0.7% RMS) and vertical (3% peak-to-peak, 0.6% RMS) beta-beats. By comparison, before any beam-based symmetrization had been attempted, the beta beating was observed to be 40% peak-to-peak (H) and 50% peak-to-peak (V). Fig. 8 ▸ shows the remaining deviation from design of the horizontal dispersion function: 2.0 mm RMS, 11 mm peak-to-peak. This is slightly larger than for the previous optics reported on by Leemann *et al.* (2018 ▸), when the dipole gradients were included in the LOCO correction.

Fig. 9 ▸ shows the remaining vertical dispersion post-correction, which has been reduced to 0.56 mm RMS and 5.2 mm peak-to-peak. At the same time, all indications are that this has been achieved along with a reduction in the betatron coupling. First, the RMS orbit response in the coupling quadrants of measured orbit response matrices (ORM) decreased to 6.3 and 6.8 µm from 12.8 and 13.0 µm, without any compensation for BPM rolls and/or coupling. Second, the observed vertical emittance could now be measured to be between 1 and 2 pm rad at the B320B diagnostic beamline^2^ (Breunlin & Andersson, 2016*a* ▸). This should be compared with the case without any skew corrections for which the vertical emittance was sometimes measured to be as high as 20 pm rad, corresponding to an emittance ratio of 6%. The fact that the applied correction giving smallest vertical emittance corresponds to the ORM with smallest coupling quadrants gives us confidence that the BPM rolls are small in comparison with the coupling.

One way to cross-check the optics given by LOCO is to measure integrated entities such as betatron tunes, horizontal emittance and momentum compaction. Obviously, LOCO predicts extremely well the measured betatron tunes, since the closed orbits inherently carry the betatron phase advance. It has not yet been possible to measure the momentum compaction factor independently, but the horizontal emittance was determined at the diagnostic beamline B320B (Breunlin, 2016 ▸; Breunlin & Andersson, 2016*b* ▸). The measurement was carried out at low current (0.6 mA) in order to minimize intensity-related effects such as intrabeam scattering and resulted in 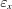 = 320 ± 18 pm rad, where the error estimate includes errors in the beam size measurement as well as the uncertainty in the determination of the electron beam optics functions. This result should be compared with the nominal value of 328 pm rad.

One long-running issue during commissioning was that changes in the fractional tune were occasionally observed between accelerator development shifts, despite identical gradient settings and correcting the RF to remove the energy shift induced by corrector magnets. These tune shifts were around 0.01. This has been traced down to orbit drifts in sextupoles generated by not using all available singular values in the response matrix inversion. In this case the final corrector settings, *i.e.* the solution converged upon by the orbit correction system, would differ depending on the starting orbit. Feed-down in the strong sextupoles of the 3 GeV ring lattice then gave the observed tune shifts.

The optics correction using LOCO ensures that the beta function values at the correctors and the BPMs, along with the total phase advance between the two points, agree well between the measurements and the model. This does not necessarily mean that the beta functions between the BPMs agree between the corrected machine and the fitted model. As such, a situation would have implications for the tuning of the non-linear optics (this is currently under investigation), in particular in light of the earlier inability of LOCO to accurately determine the dipole gradient. For this we use the well known method of engaging known local or global quadrupole gradient changes and measuring the induced betatron tune shifts. Assuming a linear optics, for example that given by LOCO, the tune shifts should be predictable, and the measured ones should agree to within the measurement accuracy. An example of this is an investigation of controlled QFE and QDE global gradient changes. A decrease of the modulus of the strength of the QFE magnet family by 0.004 m^−2^ resulted in a shift in tune by −0.0304/+0.0203 from nominal. From the optics derived from the LOCO model the same change in magnet strength was calculated to be −0.0293/+0.0204. The measurement was repeated using the QDE magnet family, resulting in a tune-shift of +0.0112/−0.0357 compared with the shift +0.0119/−0.0436 from the LOCO-derived optics. The RMS tune measurement repeatability (±0.00014) is significantly smaller than the difference between LOCO-predicted and measured tune shifts (particularly in the vertical plane at the QDEs), indicating either deviations from the optics predicted by LOCO or systematic errors in the quadrupole excitation curves. The latter has been investigated and effects from hysteresis have been excluded. If one assumes the differences are solely due to deviations from the optics given by LOCO, the systematic errors in the betatron functions predicted by LOCO range from 0.5% (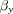 at the QFEs) to 20% (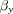 at the QDEs). A possible explanation for the larger systematic deviations in the QDEs compared with in the QFEs is that BPMs are closer to the latter and the (non-fitted) dipole gradients are closer to the former.

With the linear lattice described by Leemann *et al.* (2018 ▸), and with sextupoles and octupoles at design settings, the measured chromaticities ended up at +1 horizontally and +3 vertically, compared with the expected +1 in both planes. A 4% adjustment on the two strongest chromatic sextupole families was needed to bring the vertical chromaticity back to nominal. Scraper measurements for this lattice, without the skew quadrupole correction settings, were performed and revealed vertical and horizontal acceptances of 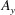 = 2.5 ± 0.2 mm mrad and 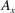 = 7.0 ± 0.4 mm mrad. More details are given by Leemann *et al.* (2018 ▸) and Sundberg (2017 ▸).

### 3.1. Dynamic aperture optimization   

After the implementation of the linear optics described in the previous section and in order to improve the ring dynamic aperture, the RCDS algorithm [robust conjugate direction search (Huang *et al.*, 2013 ▸), graciously provided by Xiaobiao Huang] was deployed. In the optimization, the lifetime of the stored beam when excited by the injection kicker was measured and the kick amplitude at which a significant reduction of lifetime occurred was used as a proxy to dynamic aperture.

The optimization procedure was run at low current (<4 mA) with a 10-bunch fill so that all bunches would obtain essentially the same kick. The algorithm was allowed to optimize using the strengths of the sextupole and octupole magnet families. In the interest of time, the algorithm changed magnet families of each magnet type by the same amounts in all 20 achromats. Singular-value decomposition (SVD) of a chromaticity response matrix containing all five sextupole families was used to construct three linear combinations of sextupole strengths which could keep the linear chromaticity constant during the optimization procedure.

Prior to the optimization, beam losses occurred at a dipole injection kicker voltage of 4–5 kV. Post-optimization process, which involved cycling the storage ring to ensure that the change was reproducible, beam loss rates >0.02 mA min^−1^ (which roughly corresponds to noise level) occurred at 5.7 kV. Despite changing the sextupole strengths in chromaticity-independent directions, some drift in chromaticity was observed. Post-optimization the chromaticity was (0.36, 1.14), compared with the initial (1.04, 0.99). The chromaticity could be corrected to (0.99, 1.02) without loss of beam resilience.

The changes made to the non-linear elements by the RCDS algorithm can be seen in Table 3 ▸.

### 3.2. Dynamic aperture measurement   

The transverse dynamic aperture of the new sextupole and octupole settings could be evaluated by looking at turn-by-turn beam losses after kicking the beam either horizontally or vertically with fast pulsed magnets. A loss rate of 1% was taken as the dynamic aperture boundary. As this measurement causes the beam to be displaced several millimetres from the BPM centres, the linear approximation often used to calculate the beam charge centre,
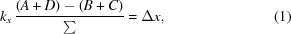


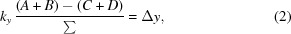
is no longer valid. In the equations above, *A*, *B*, *C* and *D* are the signal amplitudes in each of the four BPM buttons, 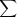 is the sum of all BPM button signal amplitudes and 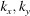 are calibration constants. A boundary element method (Stella, 1997 ▸) was therefore used to calculate the beam position from the four BPM button signals, taking into account non-linearities.

From the turn-by-turn data, the beam intensity loss and the beam amplitude could be monitored simultaneously for many turns. The loss of intensity observed is assumed to be due to the dynamic aperture of the lattice. As there are no magnetic elements between the BPMs flanking the long straight sections of the ring, the beam position data in these BPMs can be used to calculate the dynamic aperture in the 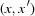 and 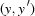 phase planes.

**Figure 10:**
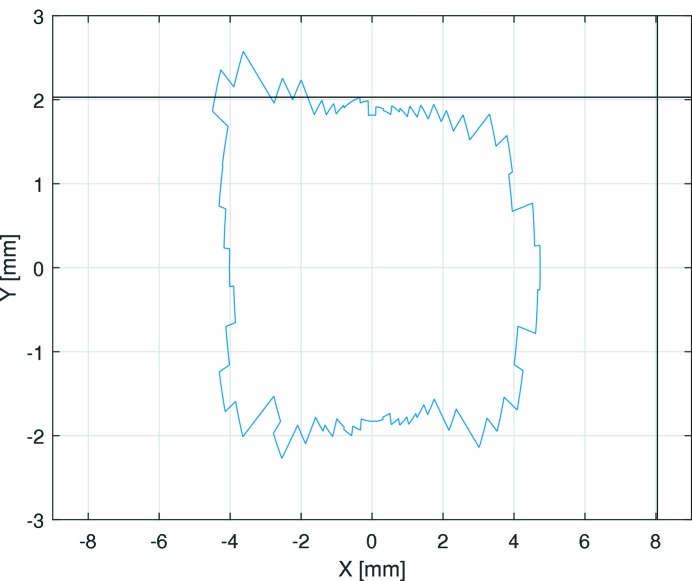
Measured transverse dynamic aperture in the middle of a long straight section. The black lines represent the result from the scraper measurements.

**Figure 11:**
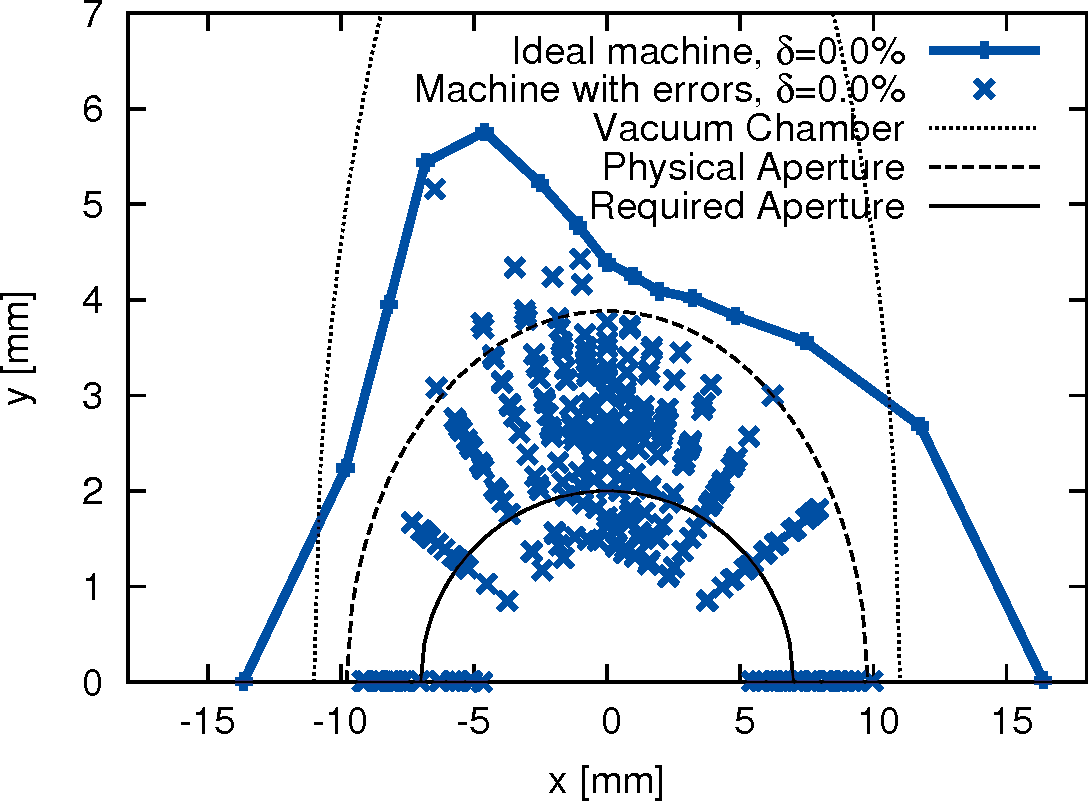
Simulated dynamic aperture in the middle of a long straight section from Leemann (2014 ▸).

**Figure 12:**
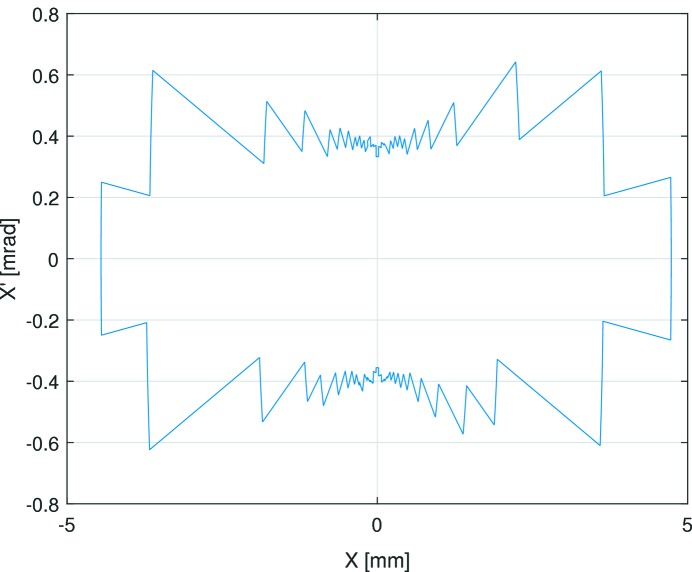
Horizontal dynamic aperture in the middle of a long straight section measured from turn-by-turn data.

The limiting transverse dynamic aperture in the middle of the straight section was found to be −4.02/+4.74 mm in the horizontal plane and −1.83/+1.86 mm in the vertical plane (see Fig. 10 ▸). For comparison, Fig. 11 ▸ shows simulation results from Leemann (2014 ▸). From LOCO, the beta functions at this location were found to be 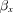 = 8.95 m and 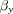 = 2.01 m. This corresponds to a horizontal acceptance of 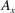 = 2.14 mm mrad and a vertical acceptance of 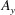 = 1.69 mm mrad. The data collection from two BPMs flanking a long straight section allows us to calculate the position and angle at the centre of the straight section (see Figs. 12 ▸ and 13 ▸). The resulting acceptances derived as the product of phase space position and angle are 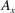 = 1.51 mm mrad and 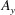 = 1.66 mm mrad. Note that this derivation is independent of any optics model. The two methods show good agreement in the vertical plane, but less so in the horizontal.

**Figure 14:**
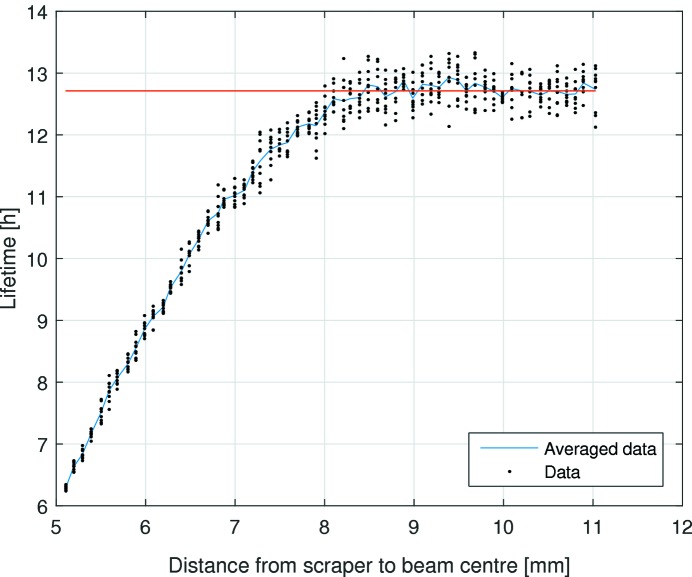
Horizontal dynamic aperture scraper measurements.

**Figure 15:**
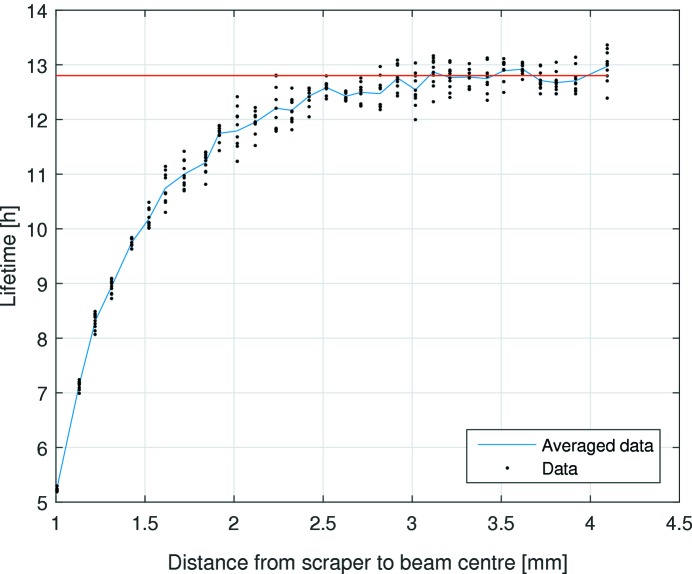
Vertical dynamic aperture scraper measurement.

Yet another method to estimate the lower limits of the dynamic aperture is to insert a horizontal and a vertical scraper while monitoring the beam lifetime. The dynamic aperture at the scraper is the distance at which the scraper starts affecting the lifetime of the beam. Measurements were performed at a beam current of 150 mA in a uniform fill. The minimum dynamic aperture was measured as 8.3 ± 0.2 mm horizontally (see Fig. 14 ▸) and 2.8 ± 0.2 mm vertically (see Fig. 15 ▸). The beta functions determined with LOCO were 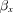 = 9.55 m at the horizontal scraper, and 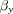 = 4.10 m at the vertical scraper. This corresponds to a horizontal and vertical acceptance of 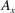 = 7.22 ± 0.35 mm mrad and 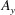 = 1.92 ± 0.28 mm mrad, respectively. Re-scaling the results of the scraper measurement to the centre of the long straight results in a dynamic aperture of 8.04 ± 0.20 mm horizontally and 2.03 ± 0.14 mm vertically.

The turn-by-turn and scraper dynamic aperture measurements are in good agreement in the vertical plane, but give significantly different values in the horizontal plane. This discrepancy is still under investigation at the time of writing; a plausible cause is decoherence of the transverse oscillations.

## 4. Coherent collective instabilities   

### 4.1. Single-bunch   

**Figure 16:**
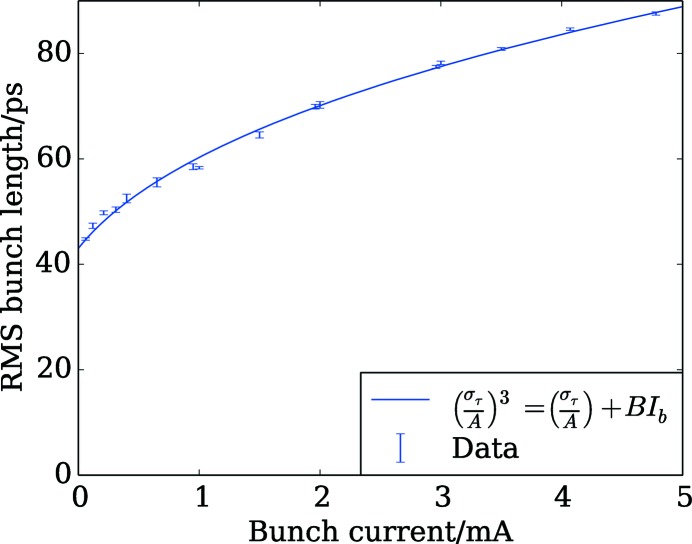
Bunch length as measured at different single-bunch currents. A cube-root curve of the form shown in the legend (with fit parameters *A* and *B*) has been fitted to the data.

Characterization of the MAX IV 3 GeV ring resistive and reactive impedance was attempted in all three planes. Where possible, the results have been compared with the accelerator impedance model (Günzel, 2009 ▸; Klein *et al.*, 2013 ▸), which is based on electromagnetic simulations of the vacuum components using *GdfidL* (Brunns, 2015 ▸). The resistive-wall contribution, including the effects of the 1 µm-thick NEG coating, was calculated using analytical formulae for the transverse plane (Burov & Lebedev, 2002 ▸) and *ImpedanceWake2D* (Mounet & Metral, 2009 ▸; Mounet, 2011 ▸) for the longitudinal plane. Fig. 16 ▸ shows the bunch length measured for different single-bunch currents. The measurements were performed using an optical sampling oscilloscope installed in the B320 diagnostic beamline (Andersson *et al.*, 2016 ▸). The lengthening with current is significant and follows a cube-root relation. Further investigation is required to determine how much of the lengthening can be attributed to potential-well distortion by the longitudinal reactive impedance so the results are only used to interpret other measurements described below.

The real part of the longitudinal impedance was probed by measuring the change in the synchronous phase as a function of the single-bunch current. For this, the phase detection of the bunch-by-bunch feedback system (see §4.3) was used. Since its response was found to be dependent on both bunch current and the RF voltage, the change in the RF phase required to zero its output reading was used as a measure of the synchronous-phase shift. A scan of the RF voltage was used to determine the absolute synchronous phase at zero current following the procedure described by Farias *et al.* (2001 ▸) and this corresponds well to the value expected from the theoretical energy loss and the RF voltage at the time of the measurement, 1.2 MV, which was estimated from the synchrotron frequency of 1.03 kHz.

**Figure 17:**
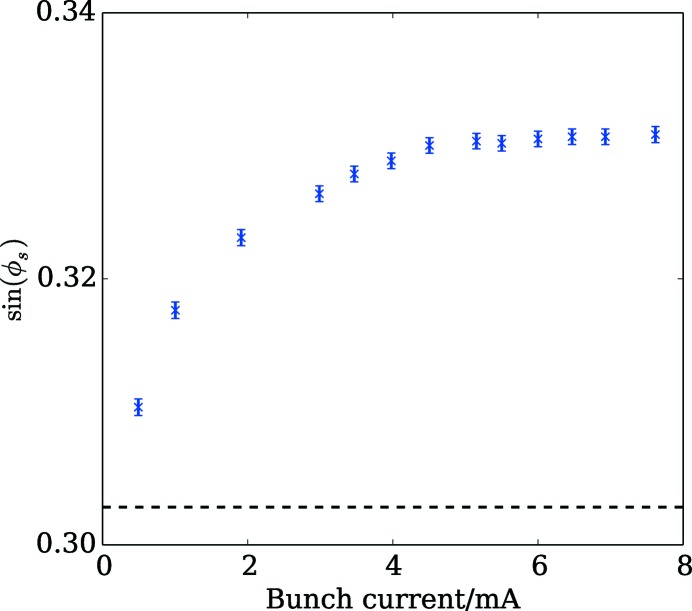
Sine of the synchronous phase measured at different bunch currents. The dotted line indicates the theoretical prediction assuming the energy loss per turn of 363.4 keV for the bare machine and the 1.2 MV amplitude of the RF.

Fig. 17 ▸ shows the sine of the synchronous phase as a function of the bunch current. The trend is far from linear due to the significant bunch lengthening over the current range. At high current, the reduction in the effective impedance due to the bunch lengthening even appears to fully compensate the increase in the bunch current within the resolution of the measurement. The loss factor at 0.75 mA was deduced from the difference in the synchronous phase with the two lowest bunch currents. This gave a value of 9800 ± 1100 mV pC^−1^, which is around three times larger than the predicted value of 3260 mV pC^−1^ for the 57 ps bunch length expected at that current.

**Figure 18:**
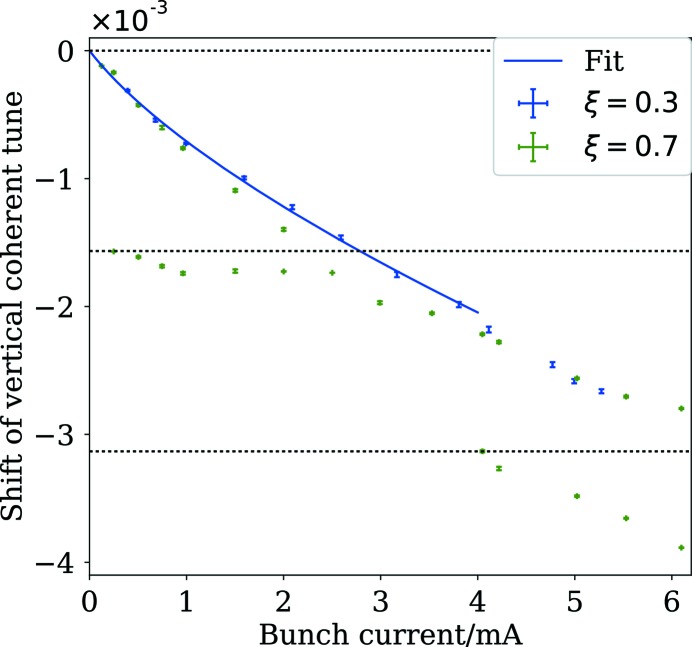
Change in the vertical tune at different bunch currents for two different values of the chromaticity. For the data at lower chromaticity, a curve is shown based on a linear fit of the tune shift against the bunch current divided by the bunch length.

Characterization of both horizontal and vertical impedance has also been performed and the results for the vertical plane are shown here. The bunch-by-bunch feedback system, and in particular its ability to drive oscillations of individual bunches across a predefined frequency range, has been used to measure the betatron tunes as a function of the bunch current. For these measurements, the bunch-by-bunch feedback system (see §4.3) was used to clean out all but one bunch in the machine. The harmonic cavities were detuned and, in any case, are not expected to have a measurable effect for such low average currents. The measurements were performed both at close-to-zero chromaticity (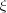 = 0.3) and at a slightly larger positive value for the chromaticity (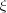 = 0.7). In the latter case, it is possible to distinguish peaks in the spectrum of the bunch motion which correspond to the azimuthal head–tail modes of lowest negative order. At the time of these measurements, two in-vacuum insertion devices were installed and were opened to their maximum gap so that their impedance could be neglected, while round dummy chambers were installed in the unused straight sections. The results are shown in Fig. 18 ▸.

The effect of the bunch-lengthening can be seen in the change in the slopes of the data at low current. The bunch current was therefore divided by the bunch length, as predicted by the fitted curve in Fig. 16 ▸, and a linear fit of the tune shift against this value was used to estimate the effective imaginary component of the vertical impedance (Chao, 1993 ▸), assuming that its change with bunch length is negligible. The four data points at highest bunch current were neglected because they appear to deviate from the expected trend, either due to a mode coupling or a non-negligible change in the effective impedance. In this way, the effective impedance is estimated at 470 ± 2 kΩ m^−1^, which is more than a factor of three larger than the 150 kΩ m^−1^ predicted using the impedance model. It is thought that one contribution to the missing impedance in all three planes is the metal-coated ceramic vacuum chambers of the injection kicker and vertical pinger magnets, which have been modelled, as for all vacuum component geometries, as a perfect conductor. A detailed investigation such as the one described by Carlà *et al.* (2016 ▸) must be carried out to identify the sources of the missing impedance so that the accuracy of the impedance model can be improved.

The detuning has also been measured in the horizontal plane. This was found to be smaller than in the vertical plane by an amount that corresponds well to the lower average beta function. The predicted effective impedance is roughly the same in both planes because of the round vacuum chamber. The effect of closing the gaps of the first two in-vacuum undulators (one to 4.5 mm and the other to 8 mm) was also investigated but it was found to be too small to be measured using the method described here. This investigation will continue with the introduction of additional insertion devices.

**Figure 19:**
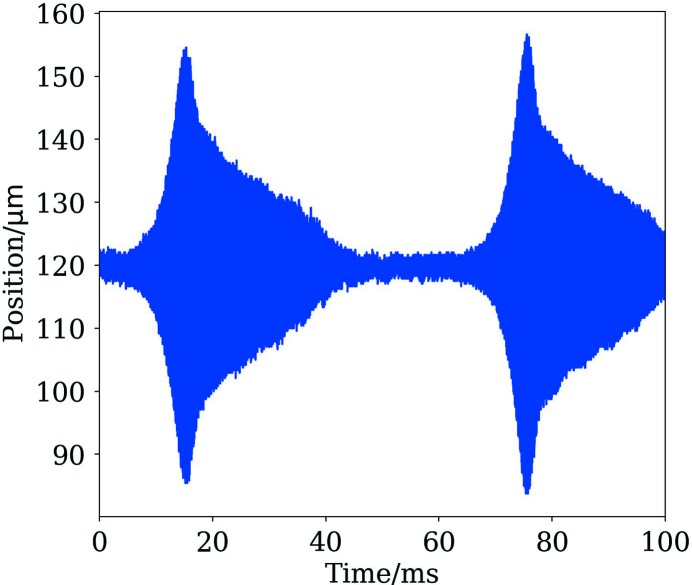
An example of the sawtooth instability observed in the MAX IV 3 GeV ring as a consequence of transverse mode coupling at low chromaticity.

The results at higher chromaticity show that the head–tail mode of azimuthal order −1 is detuned slightly in the same sense as the zero mode. The two modes can be seen to have the same frequency at a bunch current between 2 and 3 mA. At low chromaticity, this leads to a transverse mode-coupling instability and this has been observed in both planes at the expected bunch currents. The thresholds also show the expected dependence on the synchrotron tune. However, unlike at other laboratories (Koukovini-Platia *et al.*, 2017 ▸; Revol *et al.*, 2000 ▸), it is not so strong as to lead to current loss or even limit injection and can be fully damped by only a slight increase in the chromaticity (<+0.1) or by using the bunch-by-bunch feedback system. There are three probable reasons why this instability is weaker in the MAX IV 3 GeV ring than in other rings. The first is that a significant proportion of the impedance comes from multilayered chambers and is mostly reactive at low frequency where coupling to the beam is strongest and so the detuning is large but the growth time of the instability is not. The second is the bunch length, which is long compared with other laboratories and is significantly longer at high bunch currents (this is without considering any intentional Landau-cavity lengthening). Finally, the amplitude-dependent tune shift means that the instability saturates quickly and, if this is negative, it can significantly decrease the growth rate, because the tune shift from an increase in the beam size counteracts the tune shift due to the impedance. An example of the instability in the vertical plane, as observed using the bunch-by-bunch diagnostics, is shown in Fig. 19 ▸. The instability exhibits a sawtooth behaviour. This can be reproduced in macroparticle simulations where the amplitude-dependent tune shift has been included. Both in reality and in simulation it can be seen that the coherent motion of the bunch centroid decreases in amplitude much faster than the radiation damping of the beam size, which is not measured by the bunch-by-bunch feedback system and cannot be measured at the same rate with the currently available diagnostics.

### 4.2. Multibunch   

**Figure 20:**
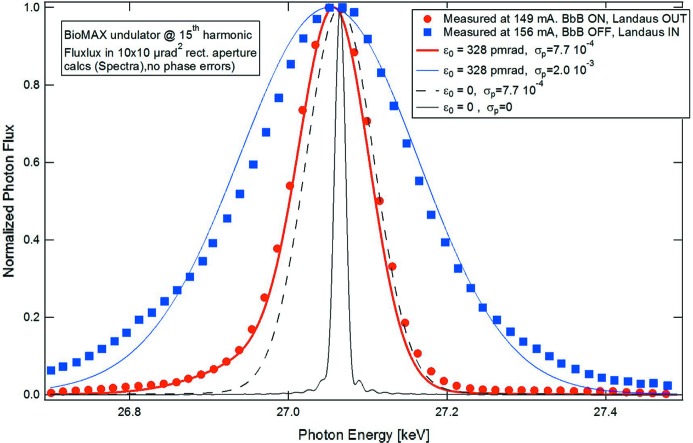
Measured and calculated undulator radiation spectral flux in a rectangular ±5 µrad aperture. Data courtesy of Thomas Ursby.

The most troublesome instabilities in the MAX IV 3 GeV ring are longitudinal coupled-bunch instabilities driven by HOMs in the main and Landau cavities. With careful temperature tuning of the main and harmonic cavities and use of the bunch-by-bunch feedback (§4.3), a longitudinally stable multibunch beam in homogeneous fill mode has been demonstrated at up to about 230 mA with the harmonic cavities detuned. The problem becomes more complex when the Landau cavities are tuned towards resonance and the anharmonicity of the RF potential increases. On one hand, this lengthens the bunches and introduces intrabunch Landau damping, but on the other hand the synchrotron tune is decreased so that the bunch-by-bunch feedback system has to provide negative feedback over a larger bandwidth that covers both the synchrotron frequency and wherever there is significant noise from the RF, around 1 kHz for example. Moreover, as the Landau cavities are tuned in towards the flat-potential condition, their HOMs are also shifted and one needs to make use of temperature tuning to maintain stability all along the Landau cavity tuning process. As a result, so far it has not been possible to fully stabilize the beam against longitudinal HOM-driven coupled-bunch instabilities using the Landau cavities alone.^3^ Tuning in of the Landau cavities does, however, lead to longer bunches and improved stability, which could be observed, for example, by measuring the width of spectral lines on the 15th harmonic of an in-vacuum undulator. Such high harmonics are quite sensitive to the electron beam energy spread, which is affected by coupled bunch oscillations. Fig. 20 ▸ shows the spectral photon flux in a pinhole geometry with a rectangular aperture of ±5 µrad from the in-vacuum undulator of the BioMAX beamline (18 mm period, 2 m length) for two different measurements: with harmonic cavities tuned in while the bunch-by-bunch feedback was off and with the bunch-by-bunch feedback on while the harmonic cavities are tuned out, both at comparable current levels. For comparison, the spectral flux calculated with the code *Spectra* (Tanaka & Kitamura, 2001 ▸) for various cases are shown. By comparing the zero emittance/zero energy spread calculated curve (black, continuous) with the zero emittance/nominal energy spread (black dashed) and nominal emittance/nominal energy spread (red) calculated curves, we verify that the width of the harmonic is mostly determined by the beam energy spread with a minor broadening coming from the finite beam emittance. The data measured at 149 mA with bunch-by-bunch feedback on and harmonic cavities detuned (red dots) agree very well with the calculated curve for nominal beam parameters (red), whereas the measured data at 156 mA with harmonic cavities tuned in and bunch-by-bunch feedback off (blue dots) is close to a calculated curve assuming the nominal emittance and 0.2% electron beam energy spread.

Since our present bunch length diagnostics is slow, we cannot differentiate between a lengthened bunch and a (slightly) longitudinally unstable bunch. For longitudinally stable conditions and in multibunch homogeneous fill mode, the largest bunch lengthening factor (*i.e.* the ratio between the measured bunch length and the natural bunch length for a given low-current synchrotron tune) was about 2 and the longest stable RMS bunch length was 98 ps. This was obtained at 108 mA by partially tuning in the harmonic cavities, *i.e.* not yet reaching flat-potential conditions.

In the transverse plane, in early stages of commissioning, ion-driven coupled-bunch instabilities were observed with a peak frequency around 10 MHz, but, after the vacuum had been conditioned for some months, higher-order mode driven instabilities were the most prevalent. At low chromaticity, these instabilities have threshold currents as low as 10–20 mA and have made the measurement of the resistive-wall instability very challenging. The latter can be observed by driving coupled-bunch mode −1 using the bunch-by-bunch feedback system and, at low chromaticity, this mode has been observed to be unstable at currents as low as 30 mA.

### 4.3. Bunch-by-bunch feedback   

**Figure 21:**
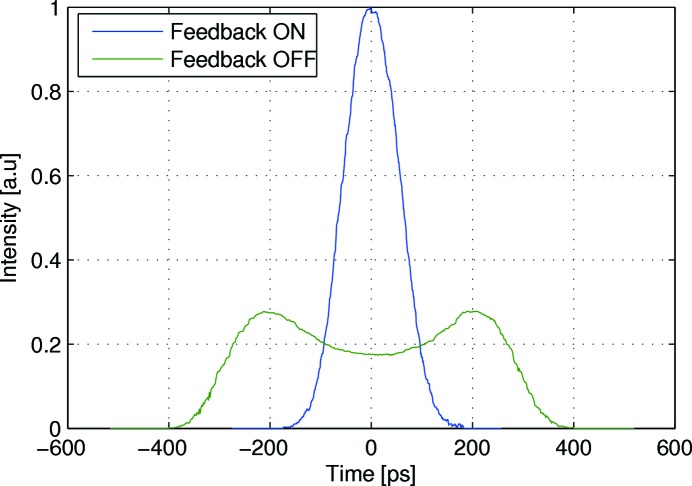
The average longitudinal bunch profile measured at a beam current of 70 mA with longitudinal feedback ON and OFF. The Landau cavities were not tuned in during these measurements, and the wider profile of the unstable beam is mainly a result of the bunches oscillating around their equilibrium, and not due to bunch lengthening. The bunch profile is obtained by measuring the temporal structure of the dipole light with an optical sampling oscilloscope (Breunlin & Andersson, 2016*a* ▸).

The 3 GeV ring has a bunch-by-bunch feedback system with Dimtel iGp12 digital signal processing units (Teytelman, 2016 ▸). The horizontal and vertical actuators are two 30 cm-long stripline kickers that are rotated 90° relative to each other. The two striplines have until the 2017 summer shutdown also simultaneously been operated as weak longitudinal actuators. This is possible by upconverting the longitudinal feedback signal to the 150–250 MHz span where the longitudinal shunt impedance of the striplines is higher. The transverse and upconverted longitudinal feedback signals are then combined and fed to the two striplines, as described by Olsson *et al.* (2017*a* ▸). With this set-up, it has been possible to keep the beam stable in all three planes at currents up to about 120 mA. Fig. 21 ▸ shows the measured bunch profile when the beam is longitudinally stable and unstable.

**Figure 22:**
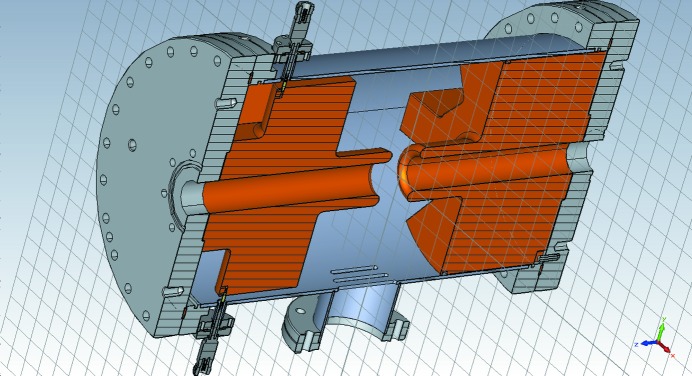
Schematic of the waveguide overloaded cavity kicker.

In October 2017, the commissioning of a waveguide overloaded cavity kicker was started (Olsson *et al.*, 2017*b* ▸,*c* ▸). The cavity is shown in Fig. 22 ▸. The centre frequencies in similar overloaded cavities might vary between 900 MHz (Wu *et al.*, 2009 ▸) and 1900 MHz (Morgan & Rehm, 2016 ▸). However, the operation span of the MAX IV cavity kicker is 600–650 MHz. The relatively low centre frequency is necessary for high kick efficiency due to the long bunches, otherwise the head and the tail of each bunch would obtain kicks with opposite directions, as explained by Olsson *et al.* (2017*c* ▸). One commercial amplifier, designed for the terrestrial UHF broadcasting band, with a maximum output RMS power of 300 W is currently feeding the cavity kicker. A second identical amplifier that doubles the total driving power will soon be added. The much higher longitudinal shunt impedance provided by the cavity, compared with the striplines, made it possible to keep the beam stable at higher currents, up to about 230 mA.

The signal processing units are controlled in EPICS, and integrated into the MAX IV control system *via* an EPICS-to-Tango gateway. Apart from applying negative feedback, the bunch-by-bunch feedback system is a comprehensive diagnostic tool. As an example, it is monitoring the betatron tunes during regular operation. Other examples of measurements performed with the system are described earlier in this section.

## 5. Beam stability   

### 5.1. General background   

In order to focus all efforts on stability at the laboratory, a Stability Task Force (STF) was created in early 2016. The mission of the STF is to achieve stable beams all the way out on the samples. Stability at the sample position means different things at different beamlines. It could be position, angle, intensity, size, coherence and any kind of frequency content. The STF is involved in all procurements at MAX IV where stability could be an issue. Some procurements are still ongoing for beamlines, but most of the work is now on characterization, handling stability issues reported at beamlines and accelerators and improvements. Work on stability started, however, much earlier, at the design stage, in an effort to ensure stable foundations for buildings, minimizing vibrations from internal sources like ventilation, cooling water systems *etc*. The main goal of the work was to reduce sensitivity to vibrations and safeguard a vibration level not very different in nature from the greenfield level. The work on stability was carried out in close collaboration with the construction company and with researchers from the Faculty of Engineering at Lund University, Department of Construction Sciences.

### 5.2. Stability philosophy   

The general philosophy for stability work at MAX IV is to use passive systems. Design of buildings, supports, tunnels, beamline and accelerator components, thermal control *etc*. are all pursuing the goal of not needing active systems as far as it goes. It is not possible to reduce ambient vibrations from the nearby highway (E22) and most other external sources. Reduction of sensitivity to vibrations is implemented by proper design of floors, girders *etc*. A coming tramline will pass relatively close to the laboratory and the tracks are vibration isolated. All internal vibration sources are, in principle, isolated using the combination of inertia and springs.

### 5.3. How was this done?   

The goals for vibration tolerances are described in the Detailed Design Report (MAX IV, 2010 ▸). The tolerances were calculated assuming no use of fast orbit feedback (FOFB). The supports are designed to have the first eigenfrequency far away from the majority of the greenfield vibration frequencies, ensuring no mechanical amplification. With an amplification factor of ten for the accelerator and assuming a tolerance of 10% of the RMS beam size as the tolerance for vibrations of the electron beam, we obtain a tolerance of 20–30 nm RMS (*f* > 5 Hz) for magnet vibrations and thus for the floors. This goal was set for the whole facility, including beamline floors.

**Figure 23:**
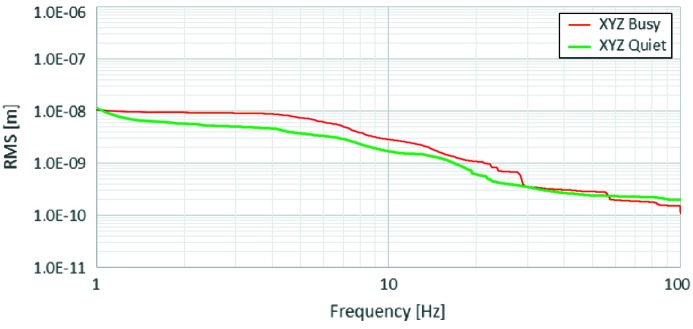
RMS floor vibrations in rush hour near the E22 highway and night time at the Balder beamline (Total amplitude XYZ, averaged over 30 min).

Fig. 23 ▸ shows typical vibration spectra for the laboratory floor nearest E22 at rush hour and at night time furthest away from E22. In general, the goal is reached, but some bursts are seen during heavy truck passages. With the known typical vibration level at the laboratory, a new goal is set for future vibration sources: new vibration sources should not add significantly to the current vibration level. This policy was followed for the design of tramline isolation and the implementation of a flywheel-based UPS system for the laboratory. Reduction of sensitivity to vibrations is achieved by increasing correlation of laboratory vibrations. The accelerator amplification factor goes down with increased correlation because increased fractions of neighbouring accelerator components are moving together in phase. The same phenomenon goes for beamlines. The goal, during the floor design phase, was as high a stiffness as possible with the given geology at the site. Various geotechnical investigations gave a simplified soil profile model, which was used in the finite-element analysis (FEA) calculations.

**Figure 24:**
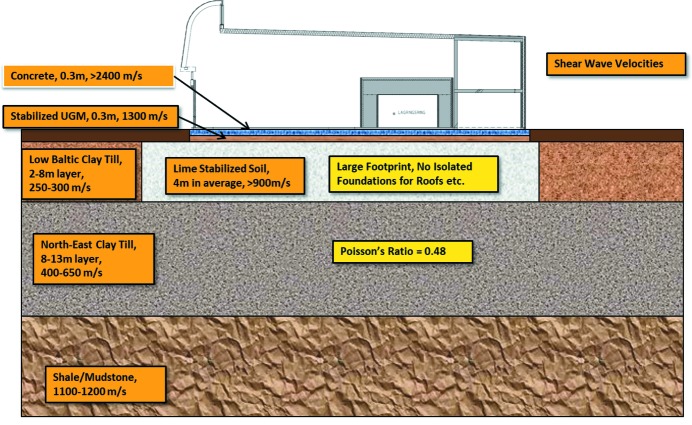
Schematic geology model and foundation design.

Fig. 24 ▸ shows a schematic cross section of the foundation and floor design. Lime-stabilized soil showed to be better than a thick concrete floor. Different layer thicknesses of lime stabilization were evaluated with FEA. A thickness of 4 m was found to be the best compromise between cost and stability. The buildings are made without using slits in the structure to isolate transportation paths or foundations for walls and roofs. The weakening of the structure would reduce stiffness and the simulations predicted increased vibration levels at the relevant frequencies. Slits only work for acoustical frequencies, since they have to be of the order of at least one-quarter of a wavelength deep.

### 5.4. Electron beam stability   

#### 5.4.1. Fast orbit motion   

Supports for accelerators and beamlines are designed to avoid system resonance over most of the greenfield vibration spectrum. The goal is a lowest eigenfrequency larger than 55 Hz. The same goal is used for monochromators and other vital structures and equipment at the laboratory. The 3 GeV ring magnets were all tested using accelerometers and a broadband shaker. The average for the first eigenmode of the different types of magnets lies between 42 Hz and 53 Hz, showing no significant floor vibration amplification.

**Figure 25:**
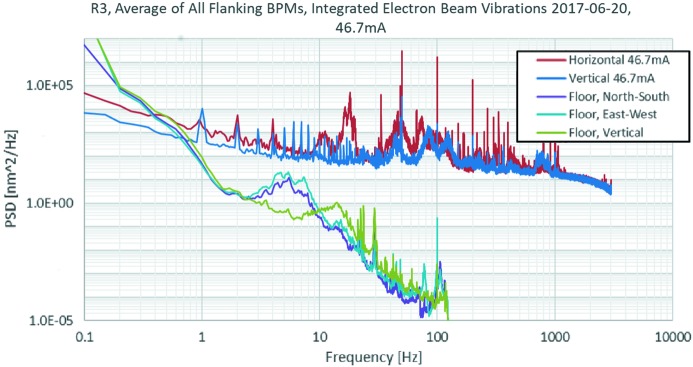
Power spectral density of electron beam vibrations and floor vibrations nearest E22.

Fig. 25 ▸ shows the power spectral density of electron beam vibrations and floor vibrations nearest E22. The majority of the greenfield vibrations were in the range 5–18 Hz. As expected, this is also the case for the floors. Due to the floor and magnet support design, we do not see a significant contribution to the electron beam vibrations in that range. More investigations are needed, but the peaks in the electron beam spectra in the range 45–125 Hz seem to have some correspondence to the first three eigenmodes of the magnet units. The source for those vibrations is probably turbulence in the cooling water.

**Figure 26:**
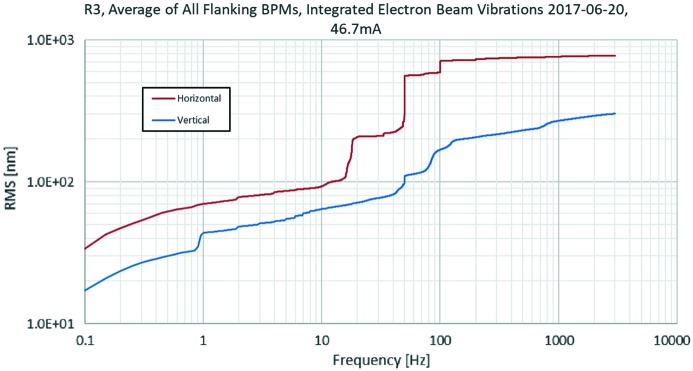
RMS integrated electron beam vibrations (square root of integrated power spectral density).

Fig. 26 ▸ shows the average of the electron beam vibration spectra for the 40 BPMs flanking a straight section in the 3 GeV ring at 46.7 mA. This is without using FOFB. The data are taken from the fast data stream provided by the Libera Brilliance+ BPM electronics (Libera Brilliance+, Instrumentation Technologies, Slovenia) at 10 kHz and are continuously monitored and archived with the Diamond fast data archiver (Abbot *et al.*, 2011 ▸).

The current goal for vertical and horizontal electron beam vibrations is 200–300 nm RMS and 5 µm RMS, respectively. Generally, the stability goal is reached, but deviations are seen when insertion devices are operated. Use of the Diamond fast archiver has allowed convenient diagnostics of beam stability: one example are 1 s periodic features which have been identified as being produced by faulty trim coil power supplies.

#### 5.4.2. Slow orbit motion   

The discussion above focused on fast beam motion related to vibrations and other high-frequency perturbations. The main tools to avoid slow beam drifts are temperature stability, top-up injection (which keeps beam current and therefore heat loads nearly constant) and a slow orbit feedback (SOFB).

The thermal stability in the ring tunnel is not fully characterized yet. Ventilation is only for replacing air in the tunnels, not for active temperature control. Air enters at two points and exits at two points in the 3 GeV ring tunnel. The inlet temperature is regulated to the exit temperature in order to minimize flow of power in or out of the tunnel due to ventilation. After reaching equilibrium, the temperature is approximately 28°C. When magnets are off, for example during maintenance, the inflow of heat from cables and coils is gone. Electrical heaters are then used to keep the flow of heat constant. The system is not perfect since the settings for the ring are changing during the first several hours after a maintenance day if magnets had to be shut off.

The SOFB uses the high-resolution 10 Hz data stream from the BPM electronics and does achieve the 10% of beam size stability goal (Tavares *et al.*, 2016 ▸; Leemann *et al.*, 2018 ▸). The current implementation as a Matlab script allows corrections at about 0.25 Hz, which has proven adequate for correcting for thermal drifts, but is not sufficient to avoid perturbations from insertion device gap/phase motions.

### 5.5. Next steps   

Maintaining beam stability during the whole life of the facility is a big challenge, since new beamlines and other installations are continuously being added, representing new potential sources of disturbances. Moreover, the stability requirements also evolve in time, as beamlines become more and more sophisticated. Tools such as the fast archiver as well as logging of temperatures and beamline performance are therefore critical to the continuous follow-up on the beam stability.

Improvements of the SOFB are ongoing to raise the correction rate up to about 10 Hz. X-ray BPMs have been recently installed and are under commissioning. The implementation of an FOFB system is also under consideration. In fact, fast orbit correctors and their cabling are already in place and power supplies for FOFB for one straight section will be installed in early 2018 for test purposes. Given the very low level of vibrations that has been obtained passively, it is, however, not yet clear that a FOFB will be necessary.

Mechanical stiffness of components for alignment will be increased in order to raise eigenfrequencies, where needed. The possibility of using mechanical active damping on structures, where the eigenfrequency goal cannot be reached, will be investigated.

## 6. Transparent top-up injection   

**Figure 27:**
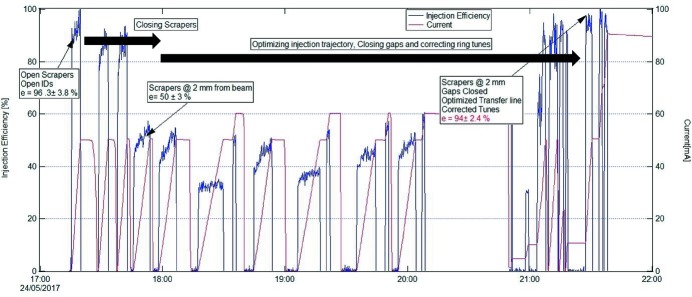
Optimization of injection efficiency with closed undulator and scraper gaps.

Automated top-up injection procedures were implemented at an early stage (see Fig. 2 ▸), before the installation of insertion devices, mainly in order to provide for a convenient way to condition the storage ring vacuum chambers. At that time, top-up was realized using a single dipole kicker magnet (Leemann, 2012*a* ▸) and accumulation at up to 20 mA min^−1^ at 2 Hz injector repetition rate and injection efficiencies^4^ above 90% were achieved through an appropriate choice of injected beam trajectory and kick strength (Tavares *et al.*, 2016 ▸). As insertion devices were installed, further trimming of the injection process was necessary to recover high efficiencies even with undulator gaps closed down to their minimum values (see Fig. 27 ▸). Additionally, in order to protect the insertion device magnets, scrapers were positioned establishing a ±2 mm vertical aperture at 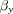 = 3.86 m, thus defining a vertical acceptance of 1.04 mm mrad.

Even though this single dipole kicker magnet injection scheme allowed top-up delivery with less than 3% dead-band and 0.25% duty cycle, the injection shots were clearly not transparent as a number of already stored bunches were kicked just as much as the injected beam and underwent betatron oscillations with amplitudes reaching several millimetres.

**Figure 28:**
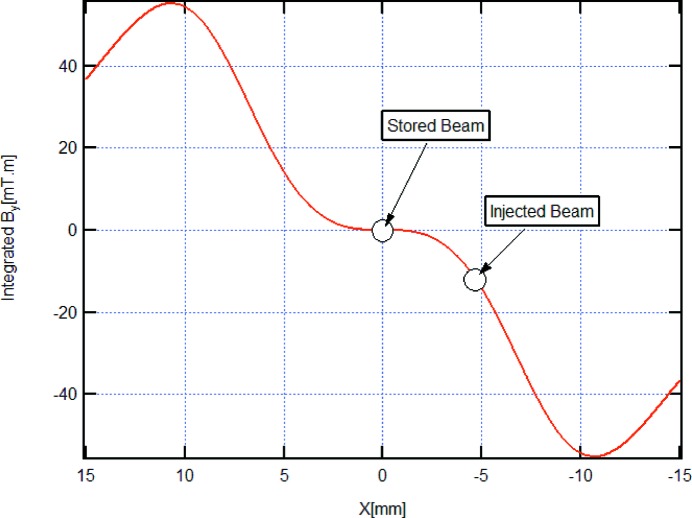
Horizontal profile of the integrated vertical field component in the MIK.

Much less disturbing top-up injection could, however, be demonstrated with the installation of a multipole injection kicker (MIK) in autumn 2017. In the MIK, the pulsed magnetic field has ideally the shape shown in Fig. 28 ▸, *i.e.* the vertical component of the magnetic field is octupole-like, being zero at the position of the stored beam and non-zero at the position of the injected beam. This is achieved with an arrangement of four current-carrying wires assembled on a precision-machined ceramic body, a concept first proposed and implemented at BESSY (Atkinson *et al.*, 2011 ▸), which, in turn, builds upon the pioneering work on sextupole pulsed magnets at KEK (Takaki *et al.*, 2010 ▸). Beam dynamics considerations for the application of the MIK concept to the MAX IV 3 GeV ring are discussed by Leemann (2012*b* ▸), whereas the engineering design and construction of the device was performed by a team led by Pierre Lebasque at SOLEIL (Lebasque, 2016 ▸) in a collaborative project involving MAX IV, SOLEIL and Helmholz Zentrum Berlin.

**Figure 29:**
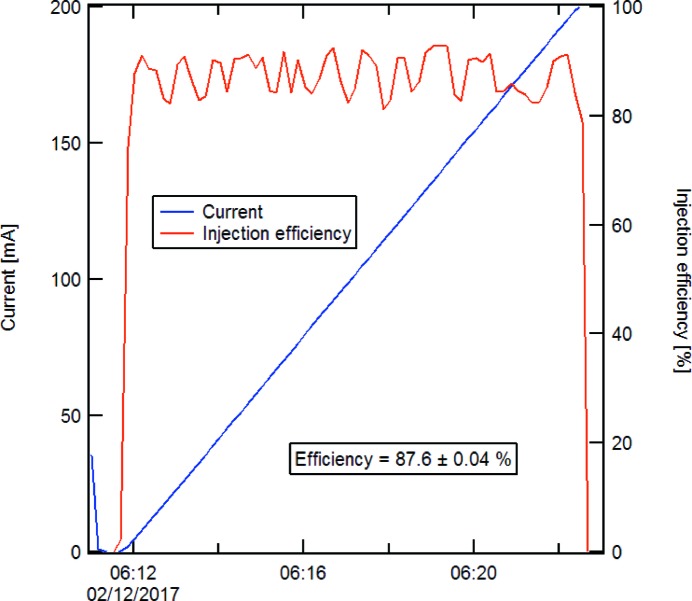
Current accumulation curve during injection with the MIK.

Beam commissioning with the MIK took advantage of the fact that the stored beam itself (still injected with the conventional single dipole kicker) could be used as a precise probe of the magnetic field distribution in the MIK. Injection with about 90% efficiency essentially independent of the amount of current in the ring was demonstrated (Fig. 29 ▸) and, in fact, the latest current record (300 mA) was also obtained with the MIK.

Once MIK injection was demonstrated, minimization of the perturbations to the stored beam was achieved by implementing local horizontal and vertical bumps and measuring the corresponding oscillation amplitudes excited by pulsing the MIK.^5^ In this experiment, a train of ten consecutive buckets spaced 10 ns from each other were filled so that all bunches experienced the same kick from the rather long MIK pulse (a half-sine wave with baseline corresponding to two turns or about 3.5 µs).

**Figure 30:**
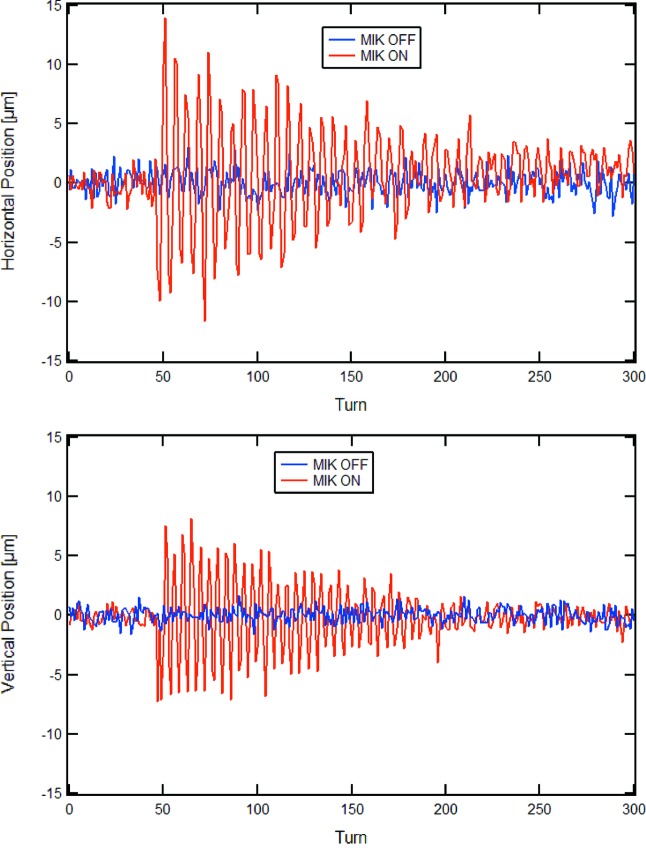
Residual stored beam horizontal (top) and vertical (bottom) oscillations excited by pulsing the MIK.

The stored current was about 20 mA and the turn-by-turn data stream from the Libera BPM electronics of one BPM in the ring was used to observe the betatron oscillations. At the position of this BPM, the horizontal and vertical betatron functions are 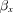 = 9.6 m and 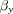 = 4.8 m, respectively. Fig. 30 ▸ shows the recorded betatron oscillations at the optimum crossing point in the MIK. The data are scaled to the centre of the long straights (with betatron functions 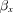 = 9.0 m and 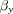 = 2.0 m, respectively), and reveal amplitudes of ±13 µm and ±8 µm in the horizontal and vertical planes, respectively.

Although these values are still large compared with the nominal beam size at the centre of the long straights (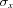 = 57 µm and 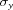 = 4 µm, assuming coupling is chosen to achieve 8 pm rad vertical emittance), they are significantly lower than those typically obtained with the more conventional four-kicker bump schemes (Loulerge, 2017 ▸; Bartholini, 2017 ▸; White, 2017 ▸) even after considerable effort in matching of the closed bump. Some residual perturbations to the stored beam were in fact expected from the mechanical measurements made prior to installation (Alexandre, 2017 ▸), which showed the positioning of the conductors to be outside tolerances. These perturbations are therefore expected to be reduced when a new MIK chamber with improved mechanics is installed in mid-2018. The tests with the prototype MIK are nevertheless a welcome confirmation that the MIK concept does lead to very good injection efficiency as well as small perturbations to the stored beam, and the MIK, since its installation, has become the standard injection kicker at the MAX IV 3 GeV ring.

## 7. Magnets   

The MAX IV 3 GeV ring magnet design is described by Johansson *et al.* (2014 ▸). The most distinctive feature of the design is the use of multifunctional compact magnet blocks housing many magnets in a common iron yoke. This allows for high eigenfrequencies of the integrated units with tight alignment tolerances within the blocks (Svensson & Johansson, 2015 ▸). The magnetic measurement results are reported by Johansson *et al.* (2015 ▸) and installation procedures are described by Åhnberg *et al.* (2016 ▸).

Operational experience with the magnet system has confirmed the soundness of the design: in fact, during early commissioning, several turns were realized without the need to excite any corrector magnets and with all magnets set to their nominal values according to magnetic measurements (Eriksson *et al.*, 2016 ▸). Apart from technical issues discovered and solved at an early stage (mostly during system tests, prior to commissioning with beam) such as short-circuited pole face strips and clogged coil cooling channels, the magnets have been running continuously without faults.

One issue related to magnet saturation was, however, somewhat puzzling at first. In fact, during the magnetic measurements, the integrated field of auxiliary windings in sextupole and octupole magnets were only measured with the main coils unpowered whereas, in real life, the main coils in those magnets are indeed powered, meaning fields are larger in the iron bringing it closer to saturation. This becomes particularly important when the field symmetries of the main and auxiliary coils are not the same, such as for example in the case of auxiliary windings producing a normal quadrupole field inside a sextupole magnet. This configuration was used to perform beam-based calibration of BPMs^6^ and, in the early experiments, a surprising dependence of the measured BPM offset with sextupole excitation current was found. In the following, we describe simulations and measurement done to understand this effect.

**Figure 31:**
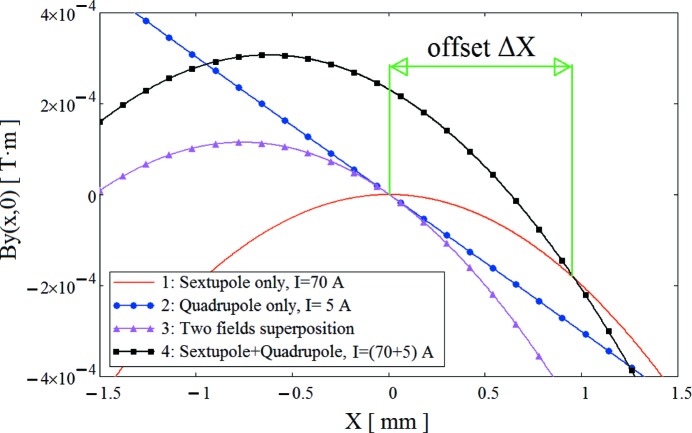
Integrated magnetic field distribution in the median plane for various modes of the coils powering.

For a pure quadrupole magnet, the value of the horizontal magnetic centre offset is defined as 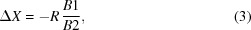
where *R* is the reference radius and *B*1 and *B*2 are, respectively, the dipole and quadrupole field components at the reference radius. In the case of a combined function magnet, with both quadrupolar and sextupolar components, the measured BPM offset value can be calculated as the distance from the geometrical centre of the magnet to a point where the magnetic field is not changed when the trim coils are powered, *i.e.* the intersection point of two curves which represent the magnetic field distributions of a pure sextupole and a combined function magnet, as shown in Fig. 31 ▸. It is clear from the figure that the non-linear behaviour of the core material makes the actual combined sextupole + quadrupole field profile different from the simple linear superpositon of the fields produced by a pure sextupole and a pure quadrupole, leading to the appearance of a horizontal offset.

**Figure 32:**
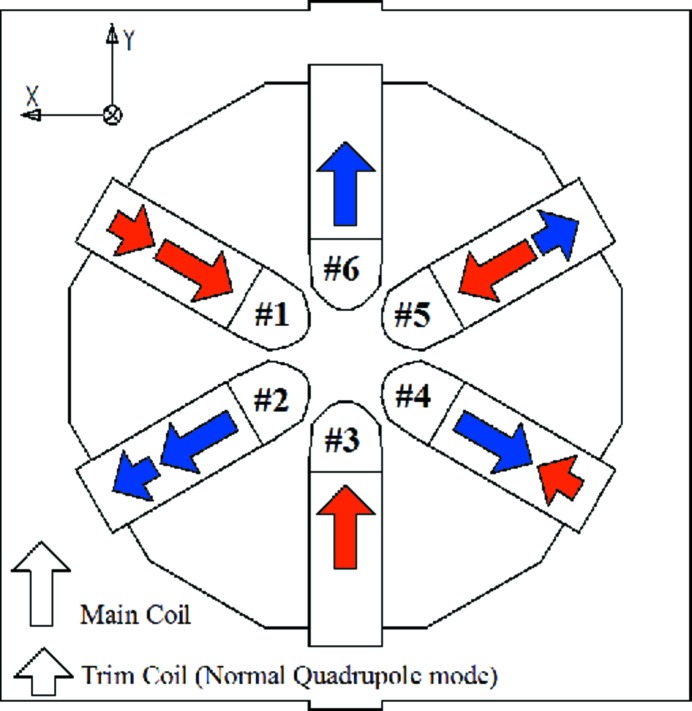
Cross section of the sextupole magnet SXFO, showing the field direction generated by the main and trim coils.

This can be better seen in Fig. 32 ▸, which shows the magnetic field direction produced by the main sextupole coils and the auxiliary (trim) coils when these are excited in the normal quadrupole mode. For such a configuration, the magnetic field direction generated by the trim coils coincides with the main coil field for the poles #1, #2 and is opposite for the poles #4, #5. As a result, poles #1 and #2 are brought closer to saturation whereas poles #3 and #4 are taken farther away from saturation breaking the sextupole symmetery and generating a horizontal offset.

**Figure 33:**
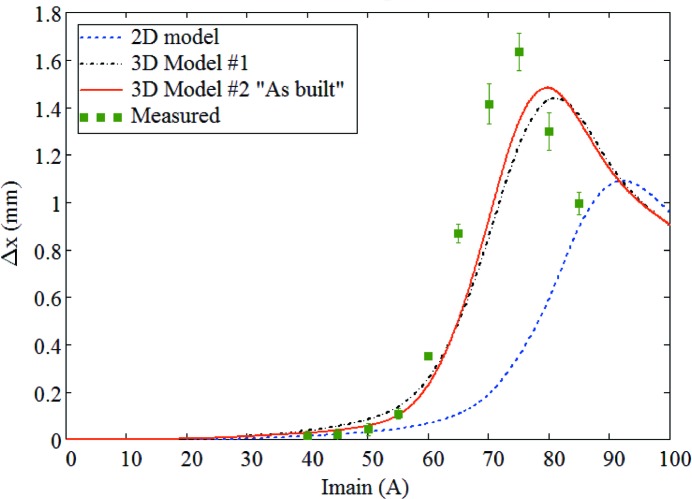
Calculated and measured horizontal offset value 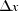 (mm) as a function of the sextupole main coil current at fixed value of the trim coil of 5 A. See Appendix *A* [App appa] for details about the various models

The results of FEA calculations (see Appendix *A* [App appa]) obtained from both two-dimensional and three-dimensional models as well as measured data are given in Fig. 33 ▸, which shows that the complete ‘as-built’ three-dimensional model #2 well predicts the magnitude and shape of offset variation with respect to the main coil current. However, according to the measurements the maximum offset value is achieved at 75 A while calculations give the peak value at 80 A. This 5 A shift between the measured and calculated data could be explained by a 10–20% difference in the *B*–*H* curves for the pole material compared with the ones assumed in the calculations.

## 8. Vacuum system   

The main distinctive feature of the vacuum system of the MAX IV 3 GeV storage ring is that the vacuum chamber walls are coated with NEG. The small magnet apertures result in a chamber with a small vacuum conductance, where the use of lumped pumps and lumped absorbers are neither effective nor practical so that NEG coating was the choice for providing the required pumping and for reducing the outgassing due to photo-stimulated desorption. NEG coating was developed at CERN (Benvenuti *et al.*, 2001 ▸) and has been used at several synchrotron facilities. Synchrotron Soleil had the previous record of the highest percentage of the chambers which are NEG-coated (56% of its overall circumference) (Herbeaux *et al.*, 2008 ▸) in a synchrotron light source, whereas 94% of the ring circumference is NEG-coated at the MAX IV 3 GeV ring. The vacuum chambers are mainly made of silver-bearing oxygen-free copper (OFS copper) as this choice of material allows the heat from the synchrotron radiation to be transmitted efficiently to the distributed absorbers. This particular alloy maintains good mechanical resistance despite the thermal cycles associated with NEG coating activation. The vacuum system design of the MAX IV 3 GeV ring is described by Al-Dmour *et al.* (2014 ▸).

Each achromat has four ion pumps, one extractor gauge and one cold cathode gauge; a few achromats are also equipped with quadrupole mass analysers (QMAs) for measuring the partial pressures. With these gauges, it is possible to measure the pressure at the extremities of the achromats; however, due to the compact lattice of the ring, there is not space to place pressure measurement devices in the middle of the achromat (over 16 m). As the pressure after the NEG activation is very low, extractor gauges were the main gauges for measuring the pressure accurately as they are able to read very low pressures (down to 10^−13^ mbar) and they are not significantly affected by the photo-electrons generated by synchrotron radiation. The reading of the pressure from the ion pumps is highly affected by the photo-electrons generated by synchrotron radiation, and their readings give only a rough indication of the pressure. The same is true for the cold cathode gauges.

**Figure 34:**
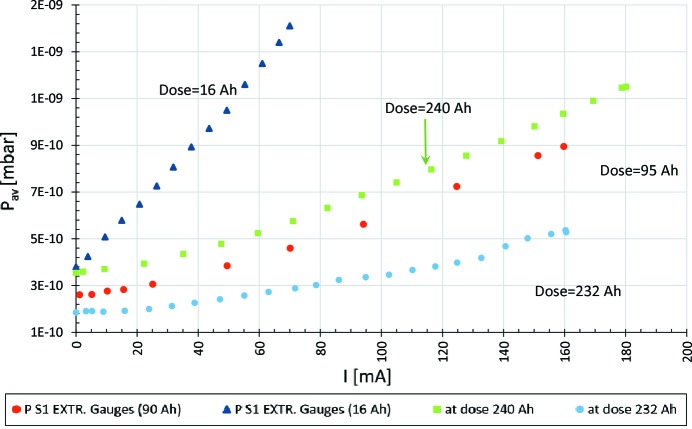
Average vacuum pressure (N_2_ equivalent) measured by the extractor gauges *versus* current at various beam doses.

At the end of the storage ring vacuum system installation (September 2015), the average pressure without beam from the extractor gauges was 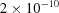
 mbar, and that from the ion pumps was 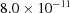
 mbar. With the first stored beam (0.1 mA), the pressure increased into the high 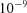
 mbar range. Since then, the vacuum conditioning is progressing and it has been observed by the average pressure reduction with the accumulated beam dose as well as by the increase of the total beam lifetime. By December 2017, the ring had an accumulated beam dose of 242 A h, and the maximum beam current was 300 mA. Since initial commissioning, there have been three main accelerator shutdowns: the first two shutdowns were dedicated to the installation of new insertion devices (no achromats were vented), while the third shutdown was dedicated to solving issues related to hot spots in the vacuum chambers and to the installation of a diagnostics beamline and, due to all these activities, three achromats had to be vented. Fig. 34 ▸ presents the average pressure (N_2_ equivalent) as measured by the extractor gauges *versus* the beam current, at different accumulated beam doses. The plot illustrates the decrease in pressure from the early commissioning stages (16 A h) to the later stage of higher accumulated doses at 95 A h and 240 A h. The beam dose was 232 A h just before the third shutdown, after which it was observed that the average pressure *versus* beam current had increased (dose 240 A h), which was expected, since three achromats had been vented during the shutdown.

**Figure 35:**
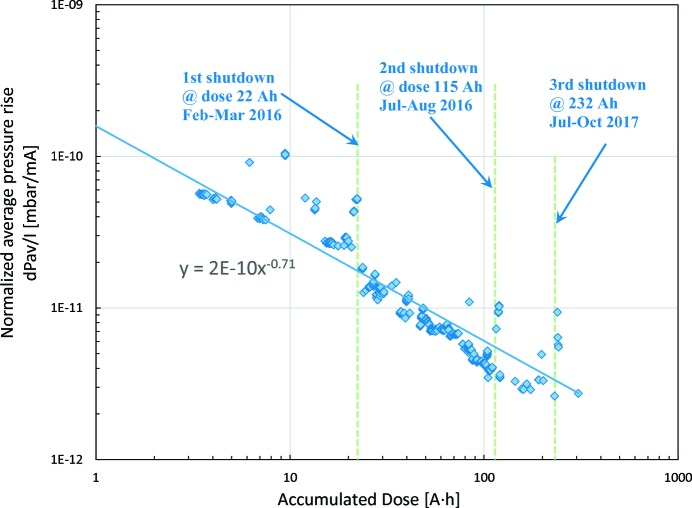
Normalized average pressure rise (mbar mA^−1^) with the accumulated beam dose.

Fig. 35 ▸ shows the normalized average pressure rise (mbar mA^−1^) as a function of the accumulated beam dose (A h). After each shutdown there was an increase of the normalized average pressure; however, with further vacuum conditioning, the average pressure recovered. The conditioning slope before the third shutdown was 0.85; however, after the shutdown the conditioning slope reduced to 0.71, a value similar to that reported at other facilities (Cox *et al.*, 2008 ▸; Herbeaux *et al.*, 2008 ▸).

**Figure 36:**
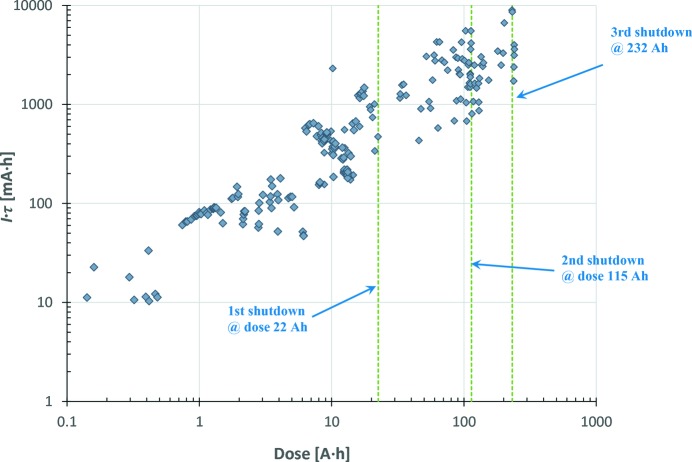
Evolution of 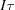 with the integrated beam dose.

Fig. 36 ▸ presents the progress in the normalized beam lifetime *versus* accumulated beam dose. The increase in the 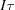 product is an indication of the beam cleaning effect and vacuum conditioning. The observed beam lifetime variations at a given level of accumulated dose may be a result of variations in beam size or bunch lengths, though this could not be confirmed due to the lack of continuously logged beam data on the bunch volume.

The approximate average gas composition (taking into account gas sensitivity for the most common gas species) from four QMA sensors around the ring at a beam current of 40 mA and an accumulated beam dose of 132 A h is: H_2_ (mass 2) 96.8%, carbon (mass 12) 0.15%, methane (mass 15 and 16) 0.7%, CO (mass 28) 2%, CO_2_ (mass 44) 0.07%, others 0.28%. Those gases are emitted due to the photo-stimulated desorption process. However, there is a clear presence of methane inside ring chambers (more than is usual in conventional vacuum systems), which is due to the fact that methane is not pumped down by the NEG coating.

As the stored beam current increased along commissioning, it was observed that some thermocouples placed on the outer walls of the vacuum chambers showed higher temperatures than the simulations indicated, mainly due to the radiation hitting the chamber wall in uncooled areas. Further investigation showed that there were several causes for this: mis­alignment of the vacuum chambers, deformation of chambers in contact with magnets due to installation errors and chamber mechanical non-conformities. During the 2017 accelerator shutdown, several of the hot spots were resolved, and the remaining hot spots shall be resolved in the 2018 summer shutdown.

Out of the 20 achromats, two were activated three times, and four were activated twice; the vacuum performance of those achromats (as measured by the pressure reading of the gauges) is similar to that of the other achromats. Although after each shutdown there is an increase in the average pressure, it has been observed that there is a fast recovery after a short vacuum conditioning period.

Simulations show that local saturation of NEG coating occurs at the areas of high outgassing, such as near the crotch absorbers, while the remaining parts of the achromat will not be saturated. However, the pressure after saturation for the crotch absorbers is in the 10^−9^ mbar range, and just 12 cm away from the crotch absorber the pressure is in the 10^−10^ mbar range (Ady *et al.*, 2014 ▸). The beam lifetime did not indicate that there is saturation for the NEG coating and the constant increase of the beam lifetime *versus* accumulated dose also indicates that there is no saturation of the NEG coating. Previous studies (Anashin *et al.*, 2004 ▸) indicated that the NEG coating has the capability to provide continuous photon-induced pumping by the getter coating even if the NEG coating is saturated with CO.

**Figure 37:**
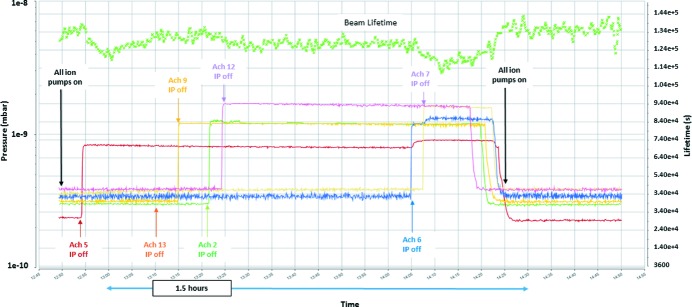
Pressure and the beam lifetime during the machine operation with some ion pumps off.

A test was performed where the ion pumps were turned off for seven achromats out of 20, while the beam was circulating at 100 mA. The main purpose of the test was to determine which pressure could be maintained by the NEG alone as well as the corresponding effect on the beam lifetime. Fig. 37 ▸ shows the beam lifetime as well as the pressure readings from the cold cathode gauges located at the achromats with the ion pumps off. An immediate reduction in the lifetime observed upon turning off the ion pumps is followed by a recovery within a few minutes, although not to the original level. When the ion pumps with a total of five achromats were off, the lifetime did not recover fast and thereafter a further decrease in the lifetime was observed when in total seven achromats were without operational ion pumps. Overall the lifetime reduced from 38 h to 32 h. No increase in the radiation level was observed outside the ring shielding wall.

**Figure 38:**
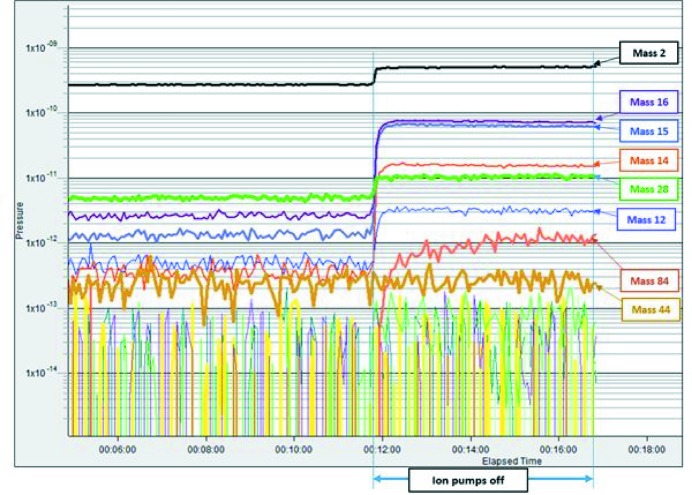
Residual gas analysis for selected masses during the machine operation with the ion pumps off.

During this test, the QMA data showed a slight increase in hydrogen, CO and carbon and no change in CO_2_. However, there was a large increase in the methane peaks. In addition, for the first time krypton (mass 84) was observed; krypton was used during the NEG coating process as the discharge gas and since it is a noble gas it is not pumped down by NEG coating. The presence of krypton, which has a high mass, could be the reason for the reduction of the beam lifetime. Fig. 38 ▸ shows the residual gas analysis for selected mass during the test. Further studies should be performed to study the vacuum performance of the NEG coating.

## 9. RF system   

The RF system for the 3 GeV ring is described by Andersson *et al.* (2011 ▸) and by Tavares *et al.* (2014*a* ▸). A main RF system of relatively low frequency, 100 MHz, has been chosen. The main cavities are made entirely of copper and are of normal-conducting capacity-loaded type. The measured shunt impedance, assuming the theoretical *R*/*Q*, amounts to 1.70 ± 0.05 MΩ per cavity [see Table 4 ▸, which is updated compared with data from Andersson *et al.* (2011 ▸) and Tavares *et al.* (2014*a* ▸), concerning the measured shunt impedances]. Six cavities are foreseen for the final operation, with estimated synchrotron radiation losses of 1 MV per turn, or 500 kW, at the design current. An RF energy acceptance of 4.5% is then reached with an overvoltage ratio of only 1.8, so the total copper losses amounts only to 159 kW. To keep a high degree of modularity, one RF station feeds each cavity. The choice fell on a combination of two commercial 60 kW solid-state amplifiers (FM band), with a 64% overall efficiency. Apart from the above-mentioned advantages of the 100 MHz system, there is the possibility to engage fast strip-line injection kickers with rise/fall times of rather moderate 10 ns. This eases the implementation of on-axis injection schemes relying on manipulation of a single bucket, reducing the dynamic aperture requirements for future magnetic lattice upgrades.

The RF system is designed with passive harmonic cavities (HCs). A double RF system is essential for achieving the storage ring performance in view of beam lifetime and beam stability, by means of lengthening the bunches and introducing additional Landau damping from increased incoherent synchrotron tune spread. Bunch lengthening is, in addition, essential to counteract intrabeam scattering and reach the design horizontal emittance at high current. An RMS bunch length of 5–6 cm is foreseen, corresponding to an elongation of about a factor of five compared with the natural bunch length. Additionally, with such an elongation the beam power spectrum is limited to low frequencies, and RF heating of vacuum components becomes less of an issue. The HCs are also normal conducting and of capacity-loaded type, mainly because this type pushes higher-order modes to relatively high frequencies compared with pill-box cavities. The fundamental mode shunt impedance was measured to be 2.70 ± 0.05 MΩ. However, with three installed HCs, the HC copper losses are of minor importance. Actually, a considerably higher total HC shunt impedance than is required for the flat-potential case (Hofmann & Myers, 1980 ▸) is installed. This is beneficial regarding the Robinson instability and allows for bunch lengthening also at lower than design currents (Tavares *et al.*, 2014*b* ▸).

Table 4 ▸ also shows the expected relevant numbers for the RF parameters during commissioning. In fact, a very similar configuration was run also during the first year of beam delivery, since the installed insertion devices were not large power consumers. The lower main cavity fields was set for reasons of the Kilpatrik limit, which is reached at 270 kV. Once the final vacuum conditions are met, the expectation is to be able to run up to 10% above the Kilpatrik limit. During the second half-year of delivery, we operated routinely four cavities at around 250 kV each, with the power coupler set to β = 2. Five cavities were installed, but one showed outgassing problems when run, even at moderate power, with stored beam. Ongoing investigations indicate a HOM is to blame and either temperature tuning or more conditioning will be needed to solve the issue.

In fact, vacuum trips in both the main and Landau cavities constituted the single most troublesome issue during early commissioning. Even though all cavities had been conditioned at high RF power before installation, many more hours of conditioning in the ring were needed before reliable operation could be achieved.

The amplitude and phase loops were, during most of the first delivery year, regulating on the cavity fields. This was necessary when careful cavity temperature tuning was initiated to combat longitudinal coupled bunch mode instabilities driven by HOMs in the cavities.

## 10. Insertion devices   

In Phase I of the MAX IV beamline projects there are five insertion devices (IDs) in the 3 GeV storage ring: two 2 m-long in-vacuum undulators (IVUs) built by industry for the BioMAX and NanoMAX beamlines, one in-vacuum wiggler (IVW) for the BALDER beamline built by SOLEIL (Marcouille *et al.*, 2013 ▸) and two 3.8 m-long elliptically polarizing undulators (EPUs) for the HIPPIE and VERITAS beamlines. The two EPUs were built in-house using the new magnetic measurement laboratory at MAX IV (Ebbeni *et al.*, 2016 ▸). The workforce of the magnetic measurements laboratory consists of a Hall probe bench covering 5.5 m magnetic length and flip coil system. Table 5 ▸ summarizes the basic parameters of the installed IDs at the 3 GeV MAX IV ring.

**Figure 39:**
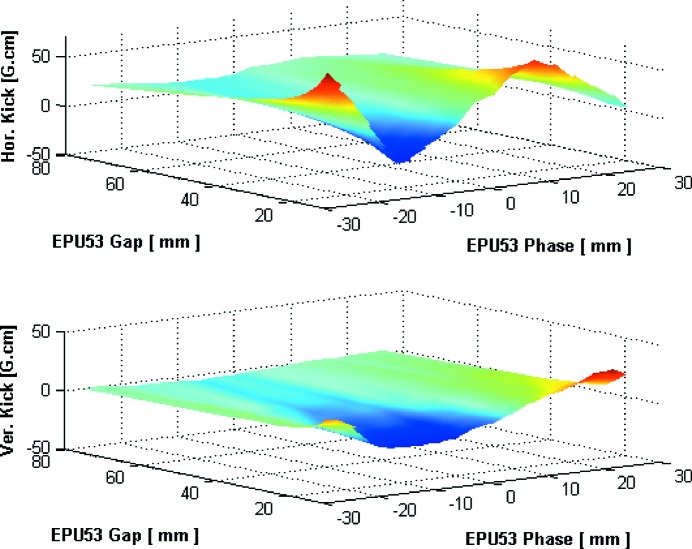
Horizontal and vertical orbit kick, in G cm, seen by the 3 GeV beam for helical mode of operation of the HIPPIE EPU.

The installation of the five IDs took place during two shutdowns in spring and fall 2016. After all necessary machine protection testing was finished, the commissioning of the different IDs started at low current (∼3 mA). Measurements of the residual field integrals were carried out to minimize orbit distortion by dedicated corrector magnets in feedforward scheme. Fig. 39 ▸ shows an example of such measurements for the HIPPIE EPU in the helical mode of operation.

Measurement of the optics distortion at different gaps, and phases for EPUs were carried out and corrections were implemented. The tune shift and linear optics distortion for the two IVUs and the EPUs were negligible. The impact of the in-vacuum wiggler is large in terms of beta-beat in the vertical plane and the approach to compensate this effect is described by Leemann & Tarawneh (2015 ▸). Preliminary measurements of the damping effect of the wiggler showed around 4% emittance reduction, confirming theoretical expectations.

**Figure 40:**
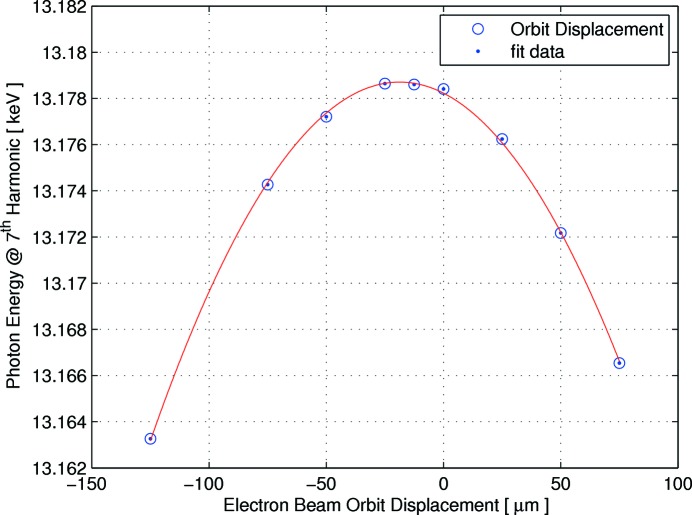
Displacement scan of the electron beam orbit at 5 mm gap inside NanoMAX IVU. The seventh harmonic energy is monitored for different offset values.

After the ID neutralization scheme from the ring side is established, each ID starts delivering for front-end and beamline commissioning. The IDs alignment was verified using the undulator harmonics for different displacements and angle of the electron beam. Furthermore an offset optimization of the electron beam inside the undulators has been achieved by monitoring the spectrum of the undulator radiation, *i.e.* by photon-beam-based alignment. Fig. 40 ▸ shows one of the displacement scans of the electron beam inside the NanoMAX IVU at gap of 5 mm and beam current of 10 mA. This alignment technique gives a precise knowledge of the magnetic centre of the ID, in this case the measurements indicates that −20 µm offset is needed for delivery from the NanoMAX undulator.

## 11. Operational experience and accelerator reliability   

2017 was the first year of regular user operations for the MAX IV 3 GeV ring. During most of the year, light delivery was performed in two shifts (08:00 to 24:00), Monday to Friday. Weekends were offered as well from November onwards. A total of 1454 h of beam time were delivered to the beamlines with 92.6% accelerator uptime. The main causes of downtime were infrastructure faults (cooling and air conditioning systems), vacuum trips during the early conditioning of beamline components and RF trips, mainly due to power cuts or brown-outs. All of these issues have been acted upon with the implementation of improved operational procedures and the ongoing installation of a rotating wheel UPS for the whole campus.

## 12. Conclusions and future perspectives   

The first ultralow-emittance storage ring based on a multibend-achromat lattice is now in user operation. The design horizontal emittance was confirmed experimentally and a vertical emittance down to 3 pm rad was demonstrated. Up to 300 mA (multibunch) and 9 mA (single-bunch) was stored in the ring and the product of beam current and lifetime has reached 7 A h after 160 A h of accumulated beam dose. Injection efficiencies in excess of 90% with transient perturbations below ±13 µm (horizontal) and ±8 µm (vertical) have been demonstrated with a multipole injection kicker. Excellent beam position stability, even without a fast orbit feedback system, was achieved. Measurements with beam indicate that the beam coupling impedance was underestimated in the design, but relevant single-bunch instability thresholds are still far above the nominal bunch currents. Multibunch instabilities, in particular longitudinal coupled-bunch modes driven by main and harmonic cavity HOMs, have shown to be the most troublesome to overcome and a bunch-by-bunch feedback system was implemented to damp those instabilities. The harmonic cavities were successfully used to provide up to a factor of two lengthening.

The first year of user operations concluded with 92.6% reliability and the main causes of beam downtime have been identified and acted upon. Full 24/7 operation will be implemented increasing the total number of delivery hours to about 4000 h in 2018.

A recurrent (but now essentially solved) issue during commissioning has been conditioning of RF cavities at high power and with stored beam.

Work planned for the near future includes further charaterization and trimming of the non-linear optics as well as further trimming of the harmonic and main cavity temperatures and bunch-by-bunch feedback to achieve higher elongation ratios at higher stored currents. Achieving the design current of 500 mA with a full suite of insertion devices will, however, require a significant upgrade of the ring RF system with duplication of the installed RF power.

In the mid- and long-term, a number of upgrades/improvements are contemplated. Higher-brightness beams can be achieved either by pushing the present 3 GeV ring lattice within the hardware constraints of the existing magnets or by more radical magnet replacements. Lattice design studies have been initiated with the long-term goal of achieving the diffraction limit at 10 keV (*i.e.* 10 pm rad horizontal emittance) within the 528 m circumference of the MAX IV 3 GeV ring tunnel (Tavares *et al.*, 2017 ▸). 

## Figures and Tables

**Figure 13:**
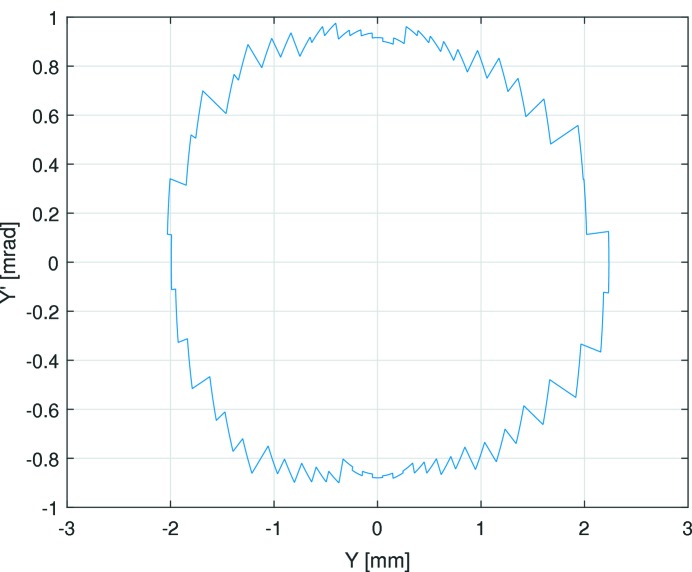
Vertical dynamic aperture in the middle of a long straight section measured from turn-by-turn data.

**Figure 41:**
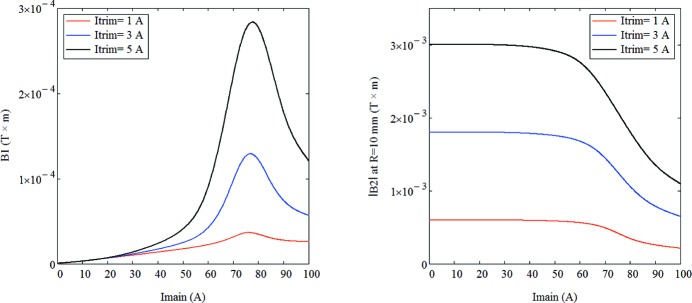
Integrated dipole component *B*1 (left) and integrated quadrupole component *B*2 at *R* = 10 mm (right) as a function of main coil current, for three values of trim coils current in normal quadruple mode.

**Figure 42:**
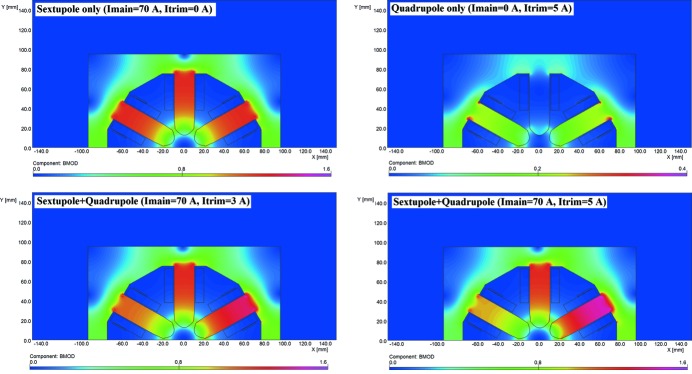
Two-dimensional model. Magnetic field distribution for various modes of the coils powering.

**Figure 43:**
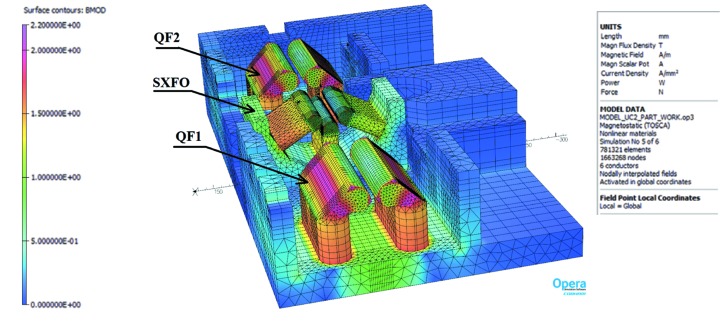
Three-dimensional model #1. Magnetic field amplitude distribution 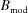 (T) on the magnet surface and mesh details.

**Figure 44:**
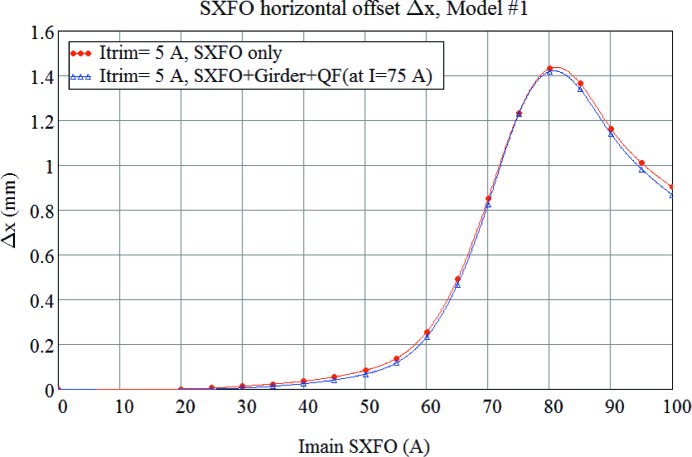
Three-dimensional model #1. Horizontal offset value 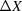
(mm) as a function of the sextupole main coil current at fixed value of the trim coil of 5 A for two model configurations.

**Figure 45:**
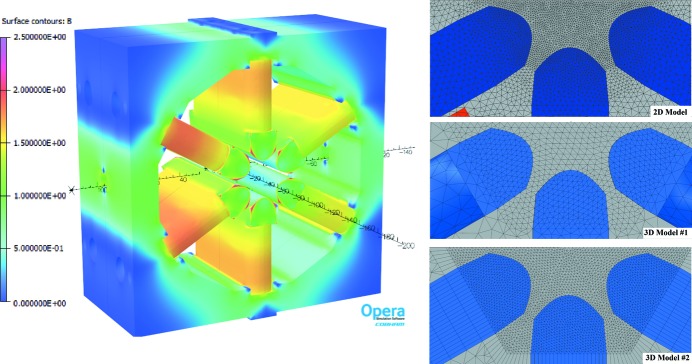
(Left) Three-dimensional model #2 with the magnetic field amplitude distribution on the magnet surface. (Right) Mesh details of two-dimensional and three-dimensional models #1 and #2.

**Table 1:** Some linac beam parameters for injection into the 3 GeV ring

Charge per shot	300 pC
Repetition rate	2 Hz
100 MHz pulses per shot	10
Injection efficiency	95%
Normalized emittance (H/V)	6 µm rad
Δ*E*/*E* in bunch train	1%

**Table 2:** Main parameters of the 3 GeV ring (bare lattice)

Circumference	528 m
Number of achromats	20
Main RF	99.931 MHz
Natural emittance	328 pm rad
Betatron tunes	42.2/16.28
Natural linear chromaticity	−50/−50.2
Linear momentum compaction	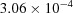
Energy spread	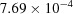
Radiated power	363.8 keV per turn

**Table 3:** Relative changes to the strengths of sextupole and octupole magnet families resulting from the RCDS algorithm

Family	Relative change (%)
SD	1.860
SDE	2.309
SFI	5.722
SFO	3.899
SFM	0.935
OXX	0.108
OXY	0.351
OYY	0.380

**Table 4:** RF system parameters

Operation phase	Commissioning	Final
Energy loss per turn (keV)	364	1000
Current (mA)	200	500
Total synchrotron radiation power (kW)	72	500
Total RF voltage (MV)	1.0	1.8
Number of cavities	4	6
Cavity voltage (kV)	250	300
Cavity shunt impedance (= *V* ^2^/2*P*) (MΩ)	1.70	1.70
Total Cu losses (kW)	74	159
Coupling, β	2.0	4.1
Number of RF stations	4	6
Minimum RF station power (with HC losses) (kW)	38	115
Total HC voltage (kV)	310	490
Number of HC	3	3
HC shunt impedance (= *V* ^2^/2*P*) (MΩ)	2.75	2.75
Total HC Cu losses (kW)	5.8	15
Bunch RMS length (mm)	60	56

**Table 5:** Main parameters of the MAX IV 3 GeV insertion devices

Beamline	HIPPIE	VERITAS	BioMAX	NanoMAX	BALDER
ID type	EPU	EPU	IVU	IVU	IVW
Period length (mm)	53	48	18	18	50
Achieved effective *K*	3.33	3.33	2.19	2.10	9
Number of periods	69	77	111	111	38
Minimum gap (mm)	11	11	4.2	4.2	4.2

**Table 6:** List of magnets and coils excitation configurations for three-dimensional model #1

	SXFO stand alone: girder and QF magnets yokes have properties of AIR			SXFO + girder + QFs		
Configuration #	1	2	3	4	5	6
*I*_main_ SXFO	(30–100) A	0 A	(30–100) A	(30–100) A	0 A	(30–100) A
*I*_trim_ SXFO	0 A	5 A	5 A	0 A	5 A	5 A
*I*_main_ QF	0 A	0 A	0 A	75 A (*I*_nom_)		

## Footnotes

1 There are two OXX magnets and two SXFO magnets in each of the 20 achromats. A detailed list of the magnets and their respective parameters are given by Johansson *et al.* (2014 ▸).

2 The B320B diagnostic beamline images visible and near-UV light emitted from a bending magnet and allows measurement of the transverse beam sizes. The source point is located at a position where the horizontal dispersion is nearly zero.

3 Note that full longitudinal stability has been achieved with Landau cavities only and no active bunch-by-bunch feedback at currents around 150 mA in the MAX IV 1.5 GeV ring.

4 Injection efficiency is defined here as the ratio of charge increase in the ring to the charge at the end of the transfer line into the ring. Typically an average over several shots is measured.

5 No attempt has been made so far to characterize or compensate for the perturbations due to MIK field gradients.

6 In the 3 GeV ring, beam-based calibration of BPMs is carried out with respect to an adjacent sextupole, instead of a quadrupole as in many other rings. The reasoning behind this is that an off-centre beam in a sextupole implies tune shifts and coupling that can be deleterious to performance, whereas an off-centre beam in a quadrupole generates an orbit distortion that can be more easily compensated for.

## Appendix A Modelling of the magnet offset

As shown in §7, exciting trim coils in quadrupole mode inside a sextupole yoke lead to the generation of a horizontal offset. In fact, even neglecting iron saturation effects, the excitation of the quadrupole trim coils breaks the sextupole symmetry and introduces higher-order () ‘non-allowed’ multipoles according to Halbach’s perturbation theory for iron dominated magnets ({Halbach, 1969 ▸). However, if iron saturation is neglected, the higher-order multipoles merely reduce the sextupole field homogeneity inside the good field region, but do not generate a horizontal offset.

The following effects which contribute to the offset due to non-linear magnetic properties of the magnetic core material were identified by numerical calculations:

(i) A normal dipole field component *B*1, which shall be zero for the pure sextupole magnet, is introduced when the trim coils are excited in normal quadrupole mode, as a result of the different saturation levels of the poles #1 and #2 as compared with the poles #3 and #4. The amplitude of this component increases as the main coil current increases, achieving its maximum at *I*_main_ ≃ 75–80 A (see Fig. 41 ▸, left). The *B*1 component decreases when the main coil current is further increased in the range from 80 A to 100 A due to the saturation of all magnet poles.

(ii) The normal quadrupole component *B*2 generated by the trim coils becomes more attenuated as the main coil current increases due to the magnet core saturation (see Fig. 41 ▸, right).

Magnetic field calculations were performed in Opera-2D/ST and Opera-3D/TOSCA programs. All simulations refer to the SXFO sextupole magnet [see Johansson *et al.* (2014 ▸) for detailed magnet parameters].

The two-dimensional model is shown in Fig. 42 ▸. Due to symmetry only half of the magnet geometry was modelled. The different field levels of the opposite poles due to the trim coils excitation are clearly visible in this figure. Since the magnet has a relatively short length (with respect to the aperture size), the fringe field in the direction of the beam axis (*z*-axis) is significant. Therefore, the two-dimensional model could not explain the magnitude of the offset effect observed in the measurements and a three-dimensional calculation was required.

Two three-dimensional models were used for the calculations. Model #1 is the SXFO sextupole magnet integrated into the magnetic girder (magnet block UC2) together with the two neighbouring QF quadrupoles (see Fig. 43 ▸). The calculations were carried out at various values of the SXFO main coil current and for different model configurations as listed in Table 6 ▸.

The calculations showed the similarity of the results obtained from the configurations #1–#3 (SXFO stand-alone) and #4–#6 (SXFO + girder and QFs), which means that the contribution from the girder and the neighbouring quadrupole magnets on the horizontal offset in the SXFO sextupole is negligible, see Fig. 44 ▸.

Results of calculations carried out for the stand-alone three-dimensional model #2 with a more detailed (‘as-built’) yoke configuration are shown in Fig. 45 ▸. Final results are shown in Fig. 33 ▸.
